# Prospective and Mendelian randomization analyses on the association of circulating fatty acid binding protein 4 (FABP-4) and risk of colorectal cancer

**DOI:** 10.1186/s12916-023-03104-1

**Published:** 2023-10-13

**Authors:** Katharina Nimptsch, Krasimira Aleksandrova, Thu Thi Pham, Nikos Papadimitriou, Jürgen Janke, Sofia Christakoudi, Alicia Heath, Anja Olsen, Anne Tjønneland, Matthias B. Schulze, Verena Katzke, Rudolf Kaaks, Bethany van Guelpen, Justin Harbs, Domenico Palli, Alessandra Macciotta, Fabrizio Pasanisi, Sandra Milena Colorado Yohar, Marcela Guevara, Pilar Amiano, Sara Grioni, Paula Gabriela Jakszyn, Jane C. Figueiredo, N. Jewel Samadder, Christopher I. Li, Victor Moreno, John D. Potter, Robert E. Schoen, Caroline Y. Um, Elisabete Weiderpass, Mazda Jenab, Marc J. Gunter, Tobias Pischon

**Affiliations:** 1 Molecular Epidemiology Research Group, Max Delbrück Center for Molecular Medicine (MDC), Berlin, Germany; 2 Department Epidemiological Methods and Etiological Research, Leibniz Institute for Prevention Research and Epidemiology, Bremen, Germany; 3 Faculty of Human and Health Sciences, University of Bremen, Bremen, Germany; 4 Charité - Universitaetsmedizin Berlin, Corporate Member of Freie Universitaet Berlin, Humboldt-Universitaet zu Berlin, Berlin, Germany; 5 Nutrition and Metabolism Branch, International Agency for Research on Cancer (IARC-WHO), Lyon, France; 6 Max-Delbrueck-Center for Molecular Medicine in the Helmholtz Association (MDC), Biobank Technology Platform, Berlin, Germany; 7 Department of Epidemiology and Biostatistics, School of Public Health, Imperial College London, London, UK; 8 Department of Inflammation Biology, School of Immunology and Microbial Sciences, King’s College London, London, UK; 9 Danish Cancer Society Research Center, Copenhagen, Denmark; 10 Department of Public Health, University of Århus, Århus, Denmark; 11 Department of Public Health, University of Copenhagen, Copenhagen, Denmark; 12 Department of Molecular Epidemiology, German Institute of Human Nutrition Potsdam-Rehbruecke, Nutehtal, Germany; 13 Institute of Nutritional Science, University of Potsdam, Nuthetal, Germany; 14 Department of Cancer Epidemiology, German Cancer Research Center (DKFZ), Heidelberg, Germany; 15 Department of Radiation Sciences, Oncology, Umeå University, Umeå, Sweden; 16 Wallenberg Centre for Molecular Medicine, Umeå University, Umeå, Sweden; 17 Cancer Risk Factors and Life-Style Epidemiology Unit, Institute for Cancer Research, Prevention and Clinical Network (ISPRO), Florence, Italy; 18 Department of Clinical and Biological Sciences, University of Turin, Turin, Italy; 19 Dipartimento di Medicina Clinica e Chirurgia, Federico II University, Naples, Italy; 20 Department of Epidemiology, Murcia Regional Health Council, IMIB-Arrixaca, Murcia, Spain; 21 CIBER Epidemiología y Salud Pública (CIBERESP), Madrid, Spain; 22 Research Group on Demography and Health, National Faculty of Public Health, University of Antioquia, Medellín, Colombia; 23 Instituto de Salud Pública de Navarra, Pamplona, 31003 Spain; 24 Navarra Institute for Health Research (IdiSNA), Pamplona, 31008 Spain; 25 Ministry of Health of the Basque Government, Sub Directorate for Public Health and Addictions of Gipuzkoa, San Sebastian, Spain; 26 Epidemiology of Chronic and Communicable Diseases Group, Biodonostia Health Research Institute, San Sebastián, Spain; 27 Epidemiology and Prevention Unit, Fondazione IRCCS Istituto Nazionale dei Tumori di Milano, Milan, 20133 Italy; 28 Unit of Nutrition and Cancer, Cancer Epidemiology Research Programme, Catalan Institute of Oncology (ICO-IDIBELL), Barcelona, Spain; 29 Blanquerna School of Health Sciences, Ramon Llull University, Barcelona, Spain; 30 Cedars-Sinai Medical Center Samuel Oschin Comprehensive Cancer Institute, Los Angeles, CA USA; 31 Division of Gastroenterology and Hepatology, Mayo Clinic Comprehensive Cancer Center, Mayo Clinic, Phoenix, AZ USA; 32 Public Health Sciences Division, Fred Hutchinson Cancer Center, Seattle, WA USA; 33 Oncology Data Analytics Program, Catalan Institute of Oncology-IDIBELL, L’Hospitalet de Llobregat, Barcelona, Spain; 34 Department of Clinical Sciences, Faculty of Medicine and Health Sciences and Universitat de Barcelona Institute of Complex Systems (UBICS), University of Barcelona (UB), L’Hospitalet de Llobregat, Barcelona, Spain; 35 Research Centre for Hauora and Health, Massey University, Wellington, New Zealand; 36 Department of Medicine and Epidemiology, University of Pittsburgh Medical Center, Pittsburgh, PA USA; 37 Department of Population Science, American Cancer Society, Atlanta, GA USA; 38 International Agency for Research on Cancer, World Health Organization, Lyon, France

**Keywords:** FABP-4, Colorectal cancer, Mendelian randomization

## Abstract

**Background:**

Fatty acid binding protein 4 (FABP-4) is a lipid-binding adipokine upregulated in obesity, which may facilitate fatty acid supply for tumor growth and promote insulin resistance and inflammation and may thus play a role in colorectal cancer (CRC) development. We aimed to investigate the association between circulating FABP-4 and CRC and to assess potential causality using a Mendelian randomization (MR) approach.

**Methods:**

The association between pre-diagnostic plasma measurements of FABP-4 and CRC risk was investigated in a nested case-control study in 1324 CRC cases and the same number of matched controls within the European Prospective Investigation into Cancer and Nutrition (EPIC) cohort. A two-sample Mendelian randomization study was conducted based on three genetic variants (1 cis, 2 trans) associated with circulating FABP-4 identified in a published genome-wide association study (discovery *n* = 20,436) and data from 58,131 CRC cases and 67,347 controls in the Genetics and Epidemiology of Colorectal Cancer Consortium, Colorectal Cancer Transdisciplinary Study, and Colon Cancer Family Registry.

**Results:**

In conditional logistic regression models adjusted for potential confounders including body size, the estimated relative risk, RR (95% confidence interval, CI) per one standard deviation, SD (8.9 ng/mL) higher FABP-4 concentration was 1.01 (0.92, 1.12) overall, 0.95 (0.80, 1.13) in men and 1.09 (0.95, 1.25) in women. Genetically determined higher FABP-4 was not associated with colorectal cancer risk (RR per FABP-4 SD was 1.10 (0.95, 1.27) overall, 1.03 (0.84, 1.26) in men and 1.21 (0.98, 1.48) in women). However, in a cis-MR approach, a statistically significant association was observed in women (RR 1.56, 1.09, 2.23) but not overall (RR 1.23, 0.97, 1.57) or in men (0.99, 0.71, 1.37).

**Conclusions:**

Taken together, these analyses provide no support for a causal role of circulating FABP-4 in the development of CRC, although the cis-MR provides some evidence for a positive association in women, which may deserve to be investigated further.

**Supplementary Information:**

The online version contains supplementary material available at 10.1186/s12916-023-03104-1.

## Background

Fatty acid binding protein 4 (FABP-4) is a lipid-binding adipokine mainly expressed in adipocytes and macrophages. FABP-4 is involved in transporting fatty acids to cellular compartments, modulating intracellular lipid metabolism, and regulating gene expression [1]. In humans, elevated FABP-4 concentrations have been associated with obesity, insulin resistance, atherosclerosis, type 2 diabetes, and metabolic syndrome [2–6]. Evidence for a causal positive association between body mass index (BMI) and FABP-4 levels has been provided by Mendelian randomization (MR) studies [7, 8]. Pro-inflammatory properties of FABP-4 have also been described [6]. FABP-4 has been shown to independently predict inflammation and fibrosis in non-alcoholic fatty liver disease (NAFLD) and may have a direct pathogenic link to disease progression [9, 10]. Based on observations in breast cancer FABP-4 has been suggested as a factor that may promote obesity-associated cancer initiation and progression [11]. FABP-4 expression in adipocytes has been reported to play a key role in the progression and metastasis of ovarian cancer by facilitating fatty acid supply for rapid tumor growth [12]. Colorectal cancer (CRC) represents another tumor in which adipocytes are an integral part of the tumor micro-environment [13], therefore circulating FABP-4 may play a role in CRC development by providing a fatty acid supply for tumor growth. In addition, FABP-4 may affect CRC development through its effects on inflammation [14] and insulin resistance [15–18], two pathways that have been demonstrated to play a role in obesity-associated CRC.

Higher FABP-4 concentrations have been observed in CRC patients than in controls in two small clinical studies from China [19, 20]. In the largest of the two studies (100 CRC cases), it was also shown that FABP-4 expression was statistically significantly higher in tumor tissues than in adjacent tissues [20]. In research focusing on colon adenocarcinoma, a higher FABP-4 expression has been observed in tumor than in adjacent tissues [21] and *FABP4* was part of an 11-gene risk score that predicts recurrence of colon adenocarcinoma [22]. Collectively, laboratory and epidemiological research suggests the potential involvement of FABP-4 in CRC development. However, evidence from prospective studies on the association between circulating FABP-4 and the risk of CRC is so far lacking. Clarifying the role of FABP-4 in CRC development is important because it may potentially serve as an obesity-associated biomarker that may help identify individuals at high risk of disease who might specifically benefit from primary or secondary prevention strategies. We hypothesized that higher FABP-4 could be positively associated with CRC risk, either directly, by facilitating fatty acid supply for tumor growth [13], or indirectly through FABP-4-related enhancement of inflammation [14] and insulin resistance [15–18] (Additional file 1: Fig. S1).

Here, we investigated the association between FABP-4 concentrations measured in baseline blood samples and subsequent risk of CRC using data from a nested case-control study in the European Prospective Investigation into Cancer and Nutrition (EPIC) cohort, stratified by sex and tumor location. In MR, genetic variants associated with circulating biomarker levels can be used to assess causal associations by circumventing common types of bias in observational studies such as residual confounding and reverse causation [23]. Thus, to further improve causal inference, we additionally conducted a two-sample MR study using data from the Genetics and Epidemiology of Colorectal Cancer Consortium, Colorectal Cancer Transdisciplinary Study, and Colon Cancer Family Registry [24, 25]. In two-sample MR, gene-exposure and gene-outcome associations are derived from non-overlapping samples [26], and a combined ratio estimate is calculated to estimate the causal association between the exposure and the outcome. We examined horizontal pleiotropy and colocalization to address the validity of MR assumptions.

## Methods

### Biomarker study

Details of the study design and methods of the prospective, multinational EPIC cohort, which has the aim of elucidating the associations between diet, lifestyle, and environmental factors with cancer and other chronic diseases, have been reported previously [27]. Between 1992 and 2000 more than 520,000 participants aged between 25 and 70 years were enrolled from 23 EPIC study centers in 10 Western European countries. The baseline examinations included standardized lifestyle, medical, and personal history questionnaires, as well as anthropometry [28] and the collection of blood samples. Participants’ habitual diet in the past year was ascertained at recruitment by validated country-specific food frequency questionnaires (FFQs), diet history, or a combination of FFQs and dietary records [27]. The EPIC cohort has been approved by the ethics review board of the International Agency for Research on Cancer (IARC, Lyon, France) as well as local review boards in each participating country.

#### Identification of colorectal cancer cases

In most participating EPIC countries, incident cancer cases were identified through record linkage with regional cancer registries (Denmark, Norway, the Netherlands, Spain, Sweden, the UK, and most of the Italian study centers). In France, Germany, Greece, and Naples (Italy), active follow-up through direct contact with the study participants or their next of kin was performed through mailed questionnaires. Self-reported cancer cases were then verified by study physicians using health insurance records, information from cancer and clinical or pathology registries, and medical records provided by treating physicians. Colorectal cancer was defined based on the International Statistical Classification of Diseases, Injury and Causes of Death (10th Revision), including tumors of the colon (C18.0–C18.7), tumors that were overlapping or unspecified (C18.8–C18.9), and tumors of the rectum (C19–C20).

#### Nested case–control study

These analyses are based on a nested case-control study (end dates between December 2001 and December 2005 across EPIC centers) of 1324 first incident CRC cases and 1324 matched controls, selected by incidence density sampling from all cohort members who were alive and free of cancer at the time of diagnosis of the index case (for technical reasons, no samples from Greece and Norway were included in the present analysis). Matching factors included sex, age at blood collection (2-month to 4-year intervals), study center, time of blood collection (± 4 h), and fasting status (< 3, 3–6, or > 6 h). Women were additionally matched on menopausal status (premenopausal, perimenopausal, postmenopausal, or surgically menopausal), on the phase of the menstrual cycle (among premenopausal women), and on the use of menopausal hormone therapy (among postmenopausal women) at the time of blood collection. Although not all matching factors (e.g., menstrual cycle) were relevant for the present study question, the nested case-control set-up was designed to be used for different biomarker studies in EPIC.

Blood samples were collected from participants at baseline according to a standardized protocol and most were stored at the International Agency for Research on Cancer (Lyon, France) in liquid nitrogen at −196 °C; exceptions were Danish samples — stored locally in nitrogen vapor at −150 °C, and Swedish samples — stored in −80 °C freezers [27]. Serum concentrations of FABP-4 were measured by enzyme-linked immunosorbent assays (BioVendor Adipocyte FABP (FABP4) Human ELISA) by BioVendor (BioVendor Laboratory Medicine, Inc.; Brno, Czech Republic). Inter-assay coefficients of variation during the laboratory analysis were 6.5%, 4.4%, and < 6.0% for high, low, and pool serum quality controls, respectively. Good reliability of FABP-4 measurements four months apart has been demonstrated using a subsample of EPIC-Potsdam [29].

Circulating concentrations of high-sensitivity C-reactive protein (hsCRP), tumor necrosis factor alpha (TNF-α), C-peptide, glycated hemoglobin (HbA1c), insulin-like growth factor 1 (IGF-1), IGF binding proteins, total cholesterol, triglycerides, high-density lipoprotein cholesterol (HDL-C), low-density lipoprotein cholesterol (LDL-C), adiponectin, leptin, soluble leptin receptor (sOB-R), resistin, neopterin, fetuin-a, 25-hydroxyvitamin D, ferric reducing ability of plasma (FRAP), and reactive oxygen metabolites (ROM) were also measured in the same study participants as previously reported [16, 17, 30–36].

#### Statistical analysis

Participants were categorized by FABP-4 concentrations in sex-specific quintiles with cut-offs based on the distribution among control participants. Baseline characteristics across sex-specific FABP-4 quintiles in control participants were examined as frequencies and proportions for categorical variables, as mean (SD) for continuous variables with approximate normal distribution, and as median (25th and 75th percentile) for skewed variables. P for trend across quintiles was calculated from generalized linear models for variables expressed as means, from the Jonkheere-Terpstra test for variables expressed as percentages, and from the Kruskal-Wallis test for variables expressed as medians. Baseline characteristics were also compared in cases versus controls, overall, and stratified by sex. *P* values for the differences between cases and controls were calculated by McNemar’s test for variables expressed as percentage, Student’s paired *t* test for variables expressed as means, and Wilcoxon’s signed rank test for variables expressed as medians. Diabetes at baseline was defined as either self-reported diabetes diagnosis or HbA1c ≥ 6.5%. A-body shape index (ABSI) was calculated with coefficients of the National Health and Nutrition Examination Survey (NHANES) [37] as follows: ABSI = waist circumference (mm) × weight (kg)^−2/3^ × height (m)^5/6^.

Spearman partial correlation coefficients (controlled for age and sex) and corresponding p-values were calculated to examine the correlation between circulating FABP-4 concentration and anthropometric measurements as well as metabolic, inflammatory, and other biomarkers, considering correlations > 0.3 as relevant.

A directed acyclic graph (DAG) was created using the DAGitty web application [38] to graphically illustrate the hypothesized causal pathways between FABP-4 and CRC risk and potentially confounding factors to be considered based on prior evidence [39]. The association between circulating FABP-4 concentration at baseline and risk of colorectal, colon, proximal colon, distal colon, or rectal cancer (overall and separately by sex) in the matched nested case-control study was investigated using conditional logistic regression analysis. Due to the incidence-density sampling, the calculated odds ratios (ORs) and 95% confidence intervals (CIs) estimate incidence rate ratios and can be interpreted as relative risks (RRs). FABP-4 in relation to CRC risk was analyzed by sex-specific quintiles as well as a continuous variable per one standard deviation (SD) in controls (8.9 ng/ml). Test for trend across quintiles was performed by entering the sex-specific quintile medians as a continuous variable into the conditional logistic regression model and evaluating its significance by using Wald’s test. The association between circulating FABP-4 and CRC risk was investigated in a crude conditional logistic regression model (thereby accounting for the matching factors only) as well as in a multivariable conditional logistic regression model with adjustment for potential confounding factors including education (none, primary school, technical/professional or secondary school, longer education including university degree, not specified), Cambridge physical activity index (inactive, moderately inactive, moderately active, active, missing) [40], smoking status and intensity (never, current (1–15, 16–25, ≥ 26 cigarettes/day, pipe/cigars/occasionally, intensity missing), former (quit ≤ 10, 11–20, ≥ 20 years ago), unknown) and alcohol intake (nondrinker, former drinker, current g/day at recruitment) (Additional file 1: Fig. S1). Adding dietary variables including total energy intake, fiber intake, fruit and vegetable intake, red meat intake, processed meat intake, and fish and shellfish intake to the model did not change results appreciably and these were therefore not included as covariables. In a separate model, we added body mass index (BMI), height, and residuals of BMI- and height-adjusted waist circumference (to avoid multicollinearity) to the multivariable model to examine whether adjustment for body size changed risk estimates. There were 122 participants with missing values on waist circumference (all from the Umeå study center) in whom the waist circumference residuals were substituted with sex-specific median values. In a sensitivity analysis, we compared associations obtained with the simple imputation for waist circumference residuals to a complete case analysis. Potential heterogeneity in the associations between FABP-4 and colorectal cancer anatomical subsite (i.e., outcome subtypes) was determined using competing risk tests [41, 42]. Heterogeneity by sex was evaluated using the Q-statistic from the inverse variance method, assuming a fixed-effect model of 1 degree of freedom [43]. Both tests for heterogeneity were conducted with the continuous estimates for FABP-4 per SD. We tested for potential non-linear associations between FABP-4 and risk of CRC using fractional polynomials [44], but there was no indication of a non-linear association from the fractional polynomials for FABP-4 and CRC overall or stratified by sex in either model (all p-values for non-linearity > 0.17).

Additional analyses were performed with exclusion of participants with diabetes at baseline (*n* = 110 cases, 8.3% of all cases; *n* = 67 controls, 5.1% of all controls) as well as CRC cases (and their matched controls) diagnosed within 2 years after recruitment (*n* = 235 cases, 17.7% of all cases) to evaluate whether our findings were influenced by preclinical disease. We tested for statistical interaction by age (< / ≥ median age 59 years), BMI (< / ≥ 30 kg/m^2^), waist circumference (< / ≥ 88 cm in women, < / ≥ 102 cm in men), and A-body shape index (ABSI, sex-specific median cut-off, < / ≥ 81.2 in men, < / ≥ 73.8 in women) as well as by sex-specific median cut-off levels of biomarkers of inflammation (CRP, < / ≥ 1910 ng/ml in men, < / ≥ 2470 ng/ml in women) or insulin resistance (C-peptide, < / ≥ 4.4 ng/ml in men, < / ≥ 3.5 ng/ml in women) or biomarkers that correlated substantially with FABP-4 (*r* > 0.3), i.e., leptin (< / ≥ 4.3 ng/ml in men, < / ≥ 14.7 ng/ml in women) and FRAP (< / ≥ 1147.5 µmol/l in men, < / ≥ 926 µmol/l in women). Statistical interaction on the multiplicative scale was evaluated by including a product term of each potential interaction factor and FABP-4 (as a continuous variable) in the multivariable model including body size and evaluating its statistical significance using the Likelihood Ratio test.

Where appropriate, we performed a causal mediation analysis for nested case–control studies using conditional logistic regression [45, 46], to investigate which proportion in the association between body fatness and CRC risk could be mediated by FABP-4, based on multivariable models adjusted for education, physical activity index, smoking intensity, and alcohol intake.

### Two-sample Mendelian randomization study

We identified four single nucleotide polymorphisms (SNPs, rs2012444; rs190775685; rs77878271; rs79389622) that were independently associated (*R*^2^ < 0.01 and *P* < 5 × 10^−8^) with FABP-4 from a genome-wide association study (GWAS) on 90 proteins including FABP-4 in 21,758 individuals from 13 cohorts of European ancestry (SCALLOP consortium) [8]. The total sample size for the GWAS on FABP-4 after subtracting missing participants due to values below the limit of detection or technical issues in each cohort was 20,436. Effect sizes are expressed as standard deviations of FABP-4 concentrations. In the same publication, it was shown in MR analyses that genetically determined FABP-4 is positively associated with BMI with intermediate evidence [8]. We calculated the variance explained by each of the SNPs from the effect allele frequency, sample size, beta estimate, and standard deviation as described previously [47]. The F-statistic was calculated according to the formula: *F* = ((*n-k*-1)/*k*)/(*R*^2^/(1-*R*^2^)), where *R*^2^ is the total proportion of the explained variance in FABP-4 by all selected genetic instruments, *k* is the number of genetic instruments and *n* is the GWAS sample size of the SNP-FABP-4 association [48]. The estimates for the association between the selected SNPs and CRC were based on data from three consortia: Genetics and Epidemiology of Colorectal Cancer Consortium (GECCO), Colorectal Cancer Transdisciplinary Study (CORECT), and Colon Cancer Family Registry (CCFR), including overall 58,131 CRC cases and 67,347 control participants [49]. In these data, one of the four selected SNPs was not available (rs190775685), so the MR analysis is based only on three SNPs, that is one cis-SNP (rs77878271, closest gene *FABP4*) and two trans-SNPs (rs2012444, closest gene *PPARG*, rs79389622, closest gene *CRB2*). We calculated the minimal detectable OR per standard deviation higher genetically predicted FABP-4 for the given sample size and proportion of variance explained by the three SNPs for a power of 0.8 [50]. The association between genetically predicted FABP-4 concentration and risk of CRC or CRC subgroups (by subsites and sex) was investigated using the fixed-effects inverse-variance-weighted method (IVW) [25]. We used MR Egger as sensitivity analysis and to investigate potential horizontal pleiotropy, i.e., the possibility that a genetic variant is associated with CRC not only through FABP-4 but also through other biological pathways, in which case the exclusion-restriction assumption of MR would be violated [26]. We repeated the MR investigation using only the cis-SNP (located near the *FABP4* gene) as an instrumental variable using the Wald ratio [51]. In addition, we scanned the *FABP4* gene region (plus/minus 100 kilobasepairs) for additional SNPs associated with FABP-4 that were correlated to a certain extent (*R*^2^ < 0.1). From this approach, one additional SNP was selected (rs2011042). As a sensitivity analysis to explore the robustness of cis MR findings, we ran a cis MR using both variants (IVW) accounting for their correlation matrix. Finally, as a complementary method to MR and to evaluate the robustness of the MR estimates, we conducted colocalization analysis [52]. This method investigates whether two traits (i.e., circulating FABP-4 and CRC) are affected by a shared or two distinct causal variants and can be applied based on summary-level genetic association data. We applied enumeration colocalization based on a Bayesian framework to detect shared causal variants [53], using the genetic region that extends 50 kilobasepairs either side of the lead *FABP4* variant. The posterior probability (PP) for the hypothesis corresponding to colocalization was calculated with the (standard) prior probability set to *p* = 10^−5^ and repeated with a relaxed prior probability of *p* = 10^−4^.

All reported *p*-values are two-sided and *P*-values < 0.05 were considered statistically significant. We applied Bonferroni-correction to account for multiple tests where appropriate. MR analyses (*MR robust)* and fractional polynomials (*fracpoly*) were performed using STATA SE 15 (StataCorp, College Station, TX, USA). The cis MR accounting for the correlation matrix, the colocalization, and the causal mediation analysis were performed in R, version 4.2.1. All other analyses were performed using SAS Version 4.3 — Graphical Software Interface — SAS Enterprise Guide (SAS Institute Inc., Cary, NC, USA).

## Results

Baseline characteristics in CRC cases versus controls showed that controls had more often a university degree, were less often physically inactive, less often current smokers, had less often diabetes, and had a lower BMI, waist circumference, and A-body shape index (ABSI, Additional file 1: Tab. S1). In women, BMI did not differ significantly between CRC cases and controls, but both waist circumference and ABSI were higher in cases than in controls (Additional file 1: Tab. S2). Median FABP-4 concentration (25th, 75th percentile) was 15.1 (11.0, 20.5) ng/ml in controls and with 15.3 (11.1, 21.3) ng/ml slightly higher in CRC cases (Additional file 1: Tab. S1). In women, median FABP-4 concentration was higher in cases (19.3 ng/ml) than in controls (18.3 ng/ml), while the contrast in men was less strong (median in cases 12.4 ng/ml, and in controls 12.1 ng/ml, Supplemental Table S2). Among controls, men had substantially lower FABP-4 concentrations (median 12.1, 25th percentile 9.0, 75th percentile 16.0 ng/ml) than women (median 18.3, 25th percentile 14.0, 75th percentile 24.5, *p*-value for sex-difference from the Kruskal-Wallis test < 0.0001), which is why we divided participants in sex-specific quintiles (cut-offs based on controls) (Additional file 1: Tab. S3). Compared with female controls, male controls had higher BMI, waist circumference and ABSI and consumed more alcohol (Additional file 1: Tab. S3). Baseline characteristics of control participants by sex-specific quintiles of FABP-4 concentrations are shown in Table 1. Mean age increased across FABP-4 quintiles. Participants in the upper quintiles were more often physically inactive than those in the lower quintiles, whereas no clear trends were observed across quintiles for education or smoking status. BMI, waist circumference, and ABSI were all increased across FABP-4 quintiles and, when comparing the upper with the lower quintiles, we observed a higher proportion of participants with diabetes. Alcohol intake, total energy, fiber, and fruit and vegetable intake were slightly decreased across FABP-4 quintiles, whereas no clear trends were observed for red and processed meat or fish intake. These observations did not differ substantially when investigating baseline characteristics in male and female control participants separately (Additional file 1: Tab. S4 and S5).

**Table 1:** Baseline characteristics by (sex-specific) quintiles of FABP-4 concentrations in control participants (*n* = 1324)

	Quintile 1	Quintile 2	Quintile 3	Quintile 4	Quintile 5	*P*-trend
Quintile ranges in men	< 8.3 ng/mL	8.3– < 10.8 ng/mL	10.8– < 13.6 ng/mL	13.6– < 17.2 ng/mL	≥ 17.2 ng/mL	
Quintile ranges in women	< 13.0 ng/mL	13.0– < 16.8 ng/mL	16.8– < 20.5 ng/mL	20.5– < 26.3 ng/mL	≥ 26.33 ng/mL	
*N*	263	268	266	265	262	
Male, *n* (%)	128 (47.9)	128 (47.6)	128 (47.8)	128 (47.6)	128 (47.9)	
Female, *n* (%)	135 (51.3)	140 (52.2)	138 (51.9)	137 (51.7)	134 (51.1)	0.93
Age, years	55.7 (7.0)	57.6 (7.1)	58.2 (7.4)	59.1 (6.2)	59.8 (6.6)	<0.0001
University degree, *n* (%)	48 (18.3)	56 (20.9)	50 (18.8)	41 (15.5)	40 (15.3)	0.12
Physically inactive, *n* (%)	40 (15.2)	50 (18.7)	73 (27.4)	61 (23.0)	71 (27.1)	0.001
Recreational and household physical activity, METs/week, median (p25, p75)	83.5 (48.5, 123.2)	78.9 (49.9, 118.7)	76.1 (42.6, 115.6)	75.7 (48.6, 115.5)	75.2 (42.0, 123.0)	0.58
Smoker, *n* (%)	75 (28.5)	61 (22.8)	57 (21.4)	69 (26.0)	66 (25.2)	0.69
Body mass index, kg/m^2^, mean (SD)	24.1 (3.1)	25.2 (3.1)	26.5 (3.4)	27.4 (3.3)	28.7 (4.3)	<0.0001
Waist circumference, cm, mean (SD)	82.6 (11.3)	85.9 (11.7)	89.1 (10.8)	91.1 (10.8)	95.4 (12.5)	< 0.0001
A-body shape index, mean (SD)	76.4 (5.6)	77.2 (6.2)	77.6 (5.7)	77.5 (5.4)	78.6 (5.5)	< 0.0001
Height, cm, mean (SD)	168 (9.0)	167 (9.1)	167 (9.3)	168 (9.8)	167 (8.9)	0.40
Diabetes at baseline, *n* (%)	7 (2.7)	9 (3.4)	10 (3.8)	10 (3.8)	11 (4.2)	0.02
Alcohol intake, g/day, median (p25, p75)	10.7 (2.1, 24.8)	7.4 (1.7, 23.6)	7.7 (1.5, 20.0)	9.2 (1.8, 24.5)	6.2 (1.5, 18.3)	0.13
Dietary factors						
Energy intake, kcal/day, median (p25, p75)	2085 (1751, 2557)	2070 (1663, 2464)	2005 (1650, 2417)	2094 (1656, 2512)	1934 (1564, 2392)	0.09
Fiber, g/day, median (p25, p75)	23.8 (18.8, 30.0)	22.9 (18.0, 28.4)	22.9 (18.1, 27.2)	22.9 (18.0, 27.4)	22.6 (17.3, 26.9)	0.04
Fruits and vegetables, g/day, median (p25, p75)	401.3 (262.3, 572.8)	397.9 (282.4, 575.4)	345.7 (241.0, 546.4)	360.9 (235.2, 535.0)	331.7 (213.6, 512.0)	0.01
Red meat, g/day, median (p25, p75)	23.6 (13.0, 43.1)	24.1 (12.8, 43.3)	25.5 (12.5, 42.6)	26.9 (15.5, 45.3)	23.3 (13.3, 45.0)	0.77
Processed meat intake, g/day, median (p25, p75)	41.4 (23.9, 70.5)	44.1 (23.8, 71.5)	46.4 (25.0, 74.3)	51.6 (31.1, 75.8)	48.2 (24.7, 79.7)	0.12
Fish, g/day, median (p25, p75)	28.2 (13.5, 51.7)	25.9 (13.7, 48.7)	28.9 (16.1, 48.5)	28.9 (14.7, 52.3)	32.4 (16.4, 52.4)	0.49
FABP-4, ng/mL, median (p25, p75)	7.9 (6.5, 11.2)	13.0 (9.7, 14.8)	17.0 (12.2, 18.4)	20.7 (15.3, 23.6)	27.2 (20.3, 31.9)	< 0.0001

*P* for trend across quintiles from the generalized linear model for variables expressed as means, from the Jonckheere-Terpstra test for variables expressed as percentage, and from the Kruskal-Wallis test for variables expressed as median

Mean (SD) unless indicated otherwise; *FABP-4* Fatty acid binding protein 4, *SD* Standard deviation, *p25* 25th percentile, *p75* 75th percentile

In control participants, FABP-4 concentrations correlated statistically significantly, even after applying a Bonferroni-correction (*n* = 28 tests), with BMI and waist circumference as well as with a number of biomarkers of inflammation, metabolism, blood lipids, adipokines, antioxidative capacity, and immune function (Table 2). FABP-4 concentrations were substantially (*r* > 0.4) positively correlated with BMI and waist circumference, and weakly correlated with ABSI, whereas no correlation was observed with height. Substantial correlations were observed between FABP-4 and the adipokine leptin (*r* = 0.42) as well as with FRAP (*r* = 0.33), a biomarker of antioxidant capacity. The inflammatory marker C-reactive protein (*r* = 0.29) as well as the hyperinsulinemia marker C-peptide (*r* = 0.28) were also correlated with FABP-4.

**Table 2:** Spearman partial correlation coefficients (controlled for age and sex) between FABP-4 and body mass index, waist circumference, A-body shape index, height and blood biomarkers in control participants (*n* = 1324)

	*n*	*r*	*P*-value
Age, years	1324	0.21	< 0.0001*
**Body mass index, kg/m**^**2**^	1324	**0.44**	< 0.0001*
**Waist circumference, cm**	1263	**0.42**	< 0.0001*
A-body shape index	1263	0.12	< 0.0001*
Height, cm	1324	−0.03	0.27
High-sensitivity C-reactive protein, mg/L	758	0.29	< 0.0001*
TNF-a (pg/mL)	703	0.09	0.02
C-peptide, ng/mL	652	0.28	< 0.0001*
HbA1c, %	635	0.13	0.002
IGF1, nmol/L	649	−0.10	0.03
IGFBP1, nmol/L	1467	0.15	< 0.0001*
IGFBP2, nmol/L	1456	−0.21	< 0.0001*
IGFBP3, nmol/L	1472	0.05	0.04
IGFBP3 intact, nmol/L	1465	0.05	0.07
Total cholesterol, mmol/L	759	0.05	0.15
LDL cholesterol, mmol/L	759	0.07	0.05
HDL cholesterol, mmol/L	757	−0.14	< 0.0001*
Triglycerides, mmol/L	748	0.25	< 0.0001*
Adiponectin, μg/mL	673	−0.09	0.02
HMW-Adiponectin, μg/mL	672	−0.08	0.03
**Leptin, ng/mL**	673	**0.42**	< 0.0001*
Soluble leptin receptor, ng/mL	673	−0.23	< 0.0001*
Resistin, ng/mL	1300	0.10	0.0003*
Fetuin-a, μg/mL	1324	0.12	< 0.0001*
25-Hydroxvitamin D, nmol/L	758	−0.10	0.005
**Ferric reducing ability of plasma, µmol/l**	759	**0.33**	< 0.0001*
Reactive oxygen metabolites, Carratelli units	754	0.19	< 0.0001*
Neopterin, nmol/L	606	0.08	0.05

Correlation coefficient for age controlled for sex only. Substantial correlations (*r* > 0.3) are displayed in bold

^*^Statistically significant after Bonferroni-correction for *n* = 28 tests (*p* < 0.018)

We observed a statistically significant association between circulating FABP-4 and risk of CRC in the conditional logistic regression model accounting for the matching factors but without further adjustment (RR highest versus lowest quintile 1.32, 95% CI 1.01, 1.72; RR per SD increment in FABP-4 1.10, 95% CI 1.01, 1.21, Table 3). This association was attenuated and no longer statistically significant after multivariable adjustment (RR highest versus lowest quintile 1.26, 95% CI 0.96, 1.66; RR per SD 1.09, 95% CI 0.99, 1.19). Additional adjustment for body size (BMI, height, and BMI- and height-adjusted waist circumference residuals) further attenuated the relative risk estimates towards the null (RR highest versus lowest quintile RR 1.01, 95% CI 0.74, 1.38, RR per SD 1.01, 95% CI 0.92, 1.12). The complete case analysis excluding participants with missing waist circumference (*n* = 122) yielded the same result in the model adjusted for body size (RR per SD 1.01, 95% CI 0.91, 1.11). No statistical interaction was observed by age, BMI, waist circumference, or ABSI categories (all *p*-interactions > 0.26). Similarly, no interaction was observed by categories of C-reactive protein, leptin, or FRAP (all *p*-interaction > 0.57). However, a statistically significant interaction between FABP-4 and C-peptide was observed (*p*-interaction 0.04). Models stratified by C-peptide concentrations (sex-specific median) revealed a statistically non-significant inverse association in participants with low C-peptide (RR per SD in FABP-4 0.84, 95% CI 0.61, 1.17) and a statistically non-significant positive association in participants with high C-peptide (RR 1.04, 95% CI 0.83, 1.29).

**Table 3:** Association between baseline FABP-4 concentrations and risk of colorectal cancer (conditional logistic regression models)

FABP-4	Ca/Co	RR^a^	(95% CI)	RR^b^	(95% CI)	RR^c^	(95% CI)
*Overall (1324 cases and matched controls)*							
Quintile 1	237/263	1	Reference	1	Reference	1	Reference
Quintile 2	256/268	1.08	(0.84, 1.38)	1.08	(0.84, 1.40)	1.03	(0.79, 1.33)
Quintile 3	268/266	1.15	(0.89, 1.49)	1.14	(0.88, 1.48)	1.04	(0.79, 1.36)
Quintile 4	267/265	1.16	(0.90, 1.49)	1.12	(0.86, 1.45)	0.98	(0.74, 1.29)
Quintile 5	296/262	1.32	(1.01, 1.72)	1.26	(0.96, 1.66)	1.01	(0.74, 1.38)
p-trend			0.03		0.08		0.94
per SD of FABP-4 (8.9 ng/ml)^d^		1.10	(1.01, 1.21)	1.09	(0.99, 1.19)	1.01	(0.92, 1.12)
*Men (640 cases and matched controls)*							
Quintile 1	118/128	1	Reference	1	Reference	1	Reference
Quintile 2	119/128	1.02	(0.71, 1.45)	1	(0.68, 1.46)	0.89	(0.61, 1.32)
Quintile 3	139/128	1.20	(0.84, 1.72)	1.21	(0.83, 1.75)	1.02	(0.70, 1.50)
Quintile 4	127/128	1.09	(0.77, 1.56)	1.04	(0.71, 1.52)	0.85	(0.57, 1.26)
Quintile 5	137/128	1.19	(0.82, 1.73)	1.11	(0.74, 1.64)	0.76	(0.49, 1.19)
p-trend			0.35		0.65		0.21
per SD of FABP-4 (8.9 ng/ml)^d^		1.07	(0.93, 1.24)	1.07	(0.92, 1.23)	0.95	(0.80, 1.13)
*Women (684 cases and matched controls)*							
Quintile 1	119/135	1	Reference	1	Reference	1	Reference
Quintile 2	137/140	1.13	(0.80, 1.61)	1.14	(0.80, 1.64)	1.14	(0.79, 1.65)
Quintile 3	129/138	1.11	(0.77, 1.60)	1.12	(0.77, 1.64)	1.08	(0.73, 1.60)
Quintile 4	140/137	1.23	(0.85, 1.76)	1.22	(0.84, 1.79)	1.15	(0.77, 1.72)
Quintile 5	159/134	1.45	(0.99, 2.12)	1.46	(0.99, 2.17)	1.34	(0.84, 2.11)
p-trend			0.05		0.05		0.24
per SD of FABP-4 (8.9 ng/ml)^d^		1.12	(1.00, 1.26)	1.12	(1.00, 1.26)	1.09	(0.95, 1.25)
p-heterogeneity by sex			0.62		0.57		0.24

*FABP-4* Fatty acid binding protein 4, *Ca/Co* Numbers of cases/controls, *RR* Relative risk based on estimated incidence rate ratio, *CI* Confidence interval, *SD* Standard deviation (SD calculated in controls)

^a^Conditioned on matching factors (sex, age at blood collection, study center, time of blood collection, and fasting status; in women were additionally matched on menopausal status, phase of the menstrual cycle among premenopausal women, and use of hormone replacement therapy at the time of blood collection among postmenopausal women)

^b^Additionally adjusted for education (none, primary school, technical/professional or secondary school, longer education including university degree, not specified), physical activity index (inactive, moderately inactive, moderately active, active, missing), smoking status and intensity (never, current (1–15, 16–25, 26+ cig/day, pipe/cigars/occasionally, intensity missing), former (quit ≤ 10, 11–20, 20+ years ago, unknown), alcohol intake (nondrinker, former drinker, current drinker, current g/day at baseline)

^c^Additionally adjusted for BMI, height, and BMI- and height-adjusted waist circumference residuals

^d^SD calculated in controls

In sex-stratified analyses, FABP-4 was borderline statistically significantly positively associated with CRC risk in women in the multivariable-adjusted model (RR per SD in FABP-4 1.12, 95% CI 1.00, 1.26), which was attenuated after adjustment for body size (RR 1.09, 95% CI 0.95, 1.25; Table 3). FABP-4 was not associated with CRC risk in men in either the multivariable-adjusted model (RR per SD in FABP-4 1.07, 95% CI 0.92, 1.23) or the multivariable-adjusted model including body size (0.95, 95% CI 0.80, 1.13). Adding the body size variables one by one to the multivariable-adjusted model showed that adjustment for BMI switched direction of estimates in men from (non-significant) positive to negative (data not shown). Despite the observed differential associations of FABP-4 with CRC risk in women compared to men, no significant heterogeneity by sex was observed (Table 3). After exclusion of participants with diabetes, the positive association between FABP-4 and CRC risk in women was slightly attenuated and statistically non-significant (RR in the multivariable-adjusted model per SD 1.05, 95% CI 0.92, 1.19), while after exclusion of cases diagnosed within the first 2 years of follow-up (and their matched controls) point estimates remained statistically significant (RR per SD 1.17, 95% CI 1.03, 1.34, Additional file 1, Tab. S6). In men and overall, associations were not substantially changed after exclusion of people with diabetes or cases (and matched controls) diagnosed with the first 2 years (Additional file 1, Tab. S6).

In subgroup analyses by CRC subsite and sex (Additional file 1, Tab. S7), associations were not substantially different, although associations were slightly stronger in rectal versus colon cancer, with significant heterogeneity in conditional only (*p*-heterogeneity < 0.0001) or the multivariable-adjusted (*p*-heterogeneity = 0.0002) model, but not in the model adjusted for body size (*p*-heterogeneity = 0.35).

The three GWAS-identified SNPs (rs2012444, rs77878271, rs79389622) explained together about 1% of interindividual variance in circulating FABP-4 and had an instrument strength of *F* = 65. With the given sample size and a statistical power of 80% the minimal detectable OR per SD in genetically predicted FABP-4 based on the three SNPs was 1.17. Of the three SNPs, one was located near the *FABP4* gene (cis-SNP), while the other two were located near other genes (trans-SNPs). Details of the three SNPs including effect estimates for the SNP-FABP4 and SNP-CRC association are displayed in Table 4.

**Table 4:** Overview of selected FABP-4 SNPs for Mendelian randomization analysis

SNP	Chromosome	Position	Closest gene	Cis/trans	Effect allele	Other allele	Parameters of SNP-FABP-4 association			Parameters of SNP-CRC association		
							Effect allele frequency	beta	SE	Effect allele frequency	Beta	SE
rs2012444	3	12375956	*PPARG*	Trans	T	C	0.13	0.11	0.01	0.12	−0.01	0.01
rs77878271	8	82395535	*FABP4*	Cis	A	G	0.97	0.26	0.04	0.98	0.05	0.03
rs79389622	9	126081452	*CRB2*	Trans	A	G	0.01	−0.59	0.10	0.00	−0.07	0.08

In the two-sample Mendelian randomization analysis using all three SNPs as instrumental variables, statistically non-significant positive associations with genetically predicted higher FABP-4 were observed for CRC overall (OR per one SD genetically predicted FABP-4 1.10, 95% CI 0.95, 1.27) and in women (OR 1.21, 95% CI 0.98, 1.48) but not in men (OR 1.03, 95% CI 0.84, 1.26, Table 5). Most CRC subgroups by location and sex showed non-significant positive associations (Additional file 1: Fig. S2), except for rectal cancer overall and in men, where effect estimates were in the direction of inverse associations, but confidence intervals were wide. When we used MR Egger instead of IVW, similarly statistically non-significant positive associations were observed (OR for CRC overall 1.27, 95% CI 0.97, 1.68) and there was no indication of horizontal pleiotropy for the SNPs associated with FABP-4 (p-value of pleiotropy 0.21). When we used only the cis-SNP as an instrumental variable, associations were stronger, with a statistically non-significant positive association for genetically predicted higher FABP-4 and CRC overall (OR 1.23, 95% CI 0.97, 1.57), a statistically significant positive association for CRC (OR 1.56, 95% CI 1.09, 2.23) and colon cancer (OR 1.58, 95% CI 1.05, 2.40) in women and no association for CRC in men (OR 0.99, 95% CI 0.71, 1.37, Table 5, Additional file 1: Fig. S3). In sensitivity analyses using two moderately correlated (*R*^2^ < 0.1) SNPs within the *FABP4* gene region (rs77878271 and rs2011042) accounting for the correlation matrix, results were not changed: genetically predicted higher FABP-4 was not associated with CRC overall (OR 1.18, 95% CI 0.97, 1.43) or in men (OR 0.94, 95% CI 0.72, 1.23), whereas a statistically significant positive association with CRC was observed in women (OR 1.48, 95% CI 1.12, 1.95). Applying a conservative Bonferroni-correction accounting for the number of tests in Table 5 (*n* = 12), however, the positive associations in women in the two cis-MRs did not pass the statistical significance threshold. In the IVW, MR Egger, and cis-MR with one SNP, no statistically significant heterogeneity by sex was observed, while in the cis-MR with 2 moderately correlated SNPs, there was an indication of heterogeneity by sex (*p* = 0.02). Colocalization analysis (genetic region plus/minus 50 kilobasepairs from the lead *FABP4* variant rs77878271) for overall CRC with standard prior probability (*p* = 10^−5^) revealed a posterior probability of a shared causal variant (PP4) of only 2% (Additional file 1: Tab. S8). Similarly, there was no indication of a shared causal variant for CRC in men (PP4 = 1%) or in women (PP4 = 12%). When the colocalization analysis was repeated with relaxed prior probability (*p* = 10^−4^) there was an indication of a shared causal variant of FABP-4 and CRC in women (PP4 = 58%, Additional file 1: Tab. S9). Because none of the variants in the gene region was strongly associated with CRC (minimum *p* = 0.03 overall, minimum *p* = 0.13 in men, minimum *p* = 0.005 in women), statistical power to detect colocalization was limited.

**Table 5:** Mendelian randomization estimates between genetically predicted circulating FABP-4 concentrations and colorectal cancer risk

	OR per genetically predicted one standard deviation higher FABP-4 (95% CI)							
	All SNPs (1 cis, 2 trans)				Cis-MR (1 SNP)		Cis-MR (2 SNPs)^a^	
	IVW	*p*-value	MR Egger	*p*-value	Wald ratio	*p*-value	IVW	*p*-value
CRC, overall	1.10 (0.95, 1.27)	0.20	1.27 (0.97, 1.68)	0.08	1.23 (0.97, 1.57)	0.09	1.18 (0.97, 1.43)	0.10
CRC, men	1.03 (0.84, 1.26)	0.77	1.19 (0.79, 1.78)	0.40	0.99 (0.71, 1.37)	0.94	0.94 (0.72, 1.23)	0.66
CRC, women	1.21 (0.98, 1.48)	0.08	1.46 (0.77, 2.78)	0.25	1.56 (1.09, 2.23)	0.02	1.48 (1.12, 1.95)	0.01
*P*-heterogeneity by sex		0.29		0.59		0.07		0.02

Mendelian randomization analyses of FABP-4 and risk of colorectal cancer based on a two-sample MR with SNP-FABP-4 associations for three SNPs (one cis, two trans) from SCALLOP consortium and SNP-CRC associations from GECCO, CORECT, and CCFR

Fixed-effects inverse-variance weighted estimates (IVW) or MR Egger for MR with all SNPs; Wald ratio estimates for cis-MR

*OR* Odds ratio, *CI* Confidence interval, *FABP-4* Fatty acid binding protein 4, *IVW* Inverse-variance-weighted method, *MR* Mendelian randomization

^a^Cis-MR with two moderately correlated SNPs (rs77878271 and rs2011042), accounting for correlation matrix in IVW

Given the suggestion of a positive association between FABP-4 and CRC risk in women from both the biomarker and MR analysis, we performed a causal mediation analysis for the association between waist circumference and CRC risk (BMI was not statistically significantly associated with CRC risk in women) with FABP-4 as a potential mediator using the EPIC nested case–control study data, assuming no interaction between waist circumference and FABP-4 (because there was no indication for such interaction). We found a mediated proportion of 10% (natural direct effect per 1 cm in waist circumference in women: OR 1.012; natural indirect effect through FABP-4: OR 1.001).

## Discussion

In this prospective investigation of circulating FABP-4 and CRC risk, we found overall no strong evidence for an association, although we observed a positive association in women; this, however, was attenuated after adjustment for body size. Genetically predicted higher FABP-4 was not statistically significantly associated with CRC in the polygenic MR. In cis MR analyses, no statistically significant associations were observed for CRC overall or in men, but a statistically significant positive association was observed for CRC in women, which, however, did not pass a Bonferroni correction accounting for the number of tests in the MR.

The hypothesis underpinning our study was that higher FABP-4 may be positively associated with CRC risk, which could be biologically explained by facilitating tumor growth via increased fatty acid supply [13], or FABP-4-related enhancement of inflammation [14] and insulin resistance [15–18]. The overall weak and statistically non-significant associations of measured circulating as well as genetically predicted FABP-4 and CRC do not provide strong support for circulating FABP-4 playing an important role in CRC development. However, a positive association of FABP-4 and CRC risk in women was observed in the biomarker analysis before adjustment for body size, suggesting that the positive association was largely explained by the upregulation of FABP-4 in obesity [7]. Interestingly, also in cis MR analyses, a positive association between FABP-4 and CRC risk in women was observed (with statistically significant heterogeneity by sex in the cis MR with two moderately correlated SNPs). It should be noted that the colocalization analysis with standard prior probability did not strongly support the existence of a shared causal variant of circulating FABP-4 and CRC in women, which could indicate that FABP-4 and CRC have distinct causal variants that are in linkage disequilibrium, thereby violating the MR assumptions. However, the posterior probabilities of distinct causal variants (PP3) were overall low and the associations of the variants in the genetic region with CRC were also overall low (*p* > 0.005), suggesting that the lack of evidence for colocalization may be due to low power to detect colocalization [52]. Colocalization analysis with relaxed prior probability gave an indication of a shared causal variant for FABP-4 and CRC in women. Sex-specific differences in FABP-4 in relation to CRC are plausible, as sex differences have also been observed in the association between general obesity and CRC risk [39, 54], where stronger associations have usually been observed with BMI in men than in women. Two MR studies produced conflicting results regarding sex-differences in the association of BMI with CRC risk, where the smaller one observed a positive association between genetically predicted BMI and CRC in women but not in men [55], and the larger and more recent one (using sex-specific genetic instruments) observed a stronger positive association between genetically predicted BMI and CRC in men compared with women [56]. Sex-specific differences have been observed also in the relationship of inflammatory markers with CRC risk [14, 30], where stronger associations were observed in men than in women. A more prominent role of FABP-4 for CRC in women, as observed in our study, may also be biologically plausible on the basis of the observed higher FABP-4 concentrations in women than men, and deserves further study. It should be noted, however, that we observed no statistically significant heterogeneity by sex (*p*-heterogeneity 0.57). In contrast, we observed statistically non-significant inverse associations between FABP-4 and CRC risk in men in models accounting for body size. However, in the MR analysis, there was no indication for an inverse association between genetically determined higher circulating FABP-4 and risk of CRC in men. In the MR analysis, we observed mostly non-significant positive associations between genetically predicted FABP-4 and CRC anatomical subsites, except a non-significant inverse association with rectal cancer, which is in contrast to our findings of the biomarker analysis (non-significant positive association with rectal cancer). However, the wide confidence intervals in the MR analysis point to the uncertainty of the inverse association with rectal cancer.

When we performed a causal mediation analysis for the association between waist circumference and CRC risk with FABP-4 as a potential mediator in women, we found that a rather small proportion of the association (10%) was mediated by FABP-4. Compared with biomarkers such as non-HMW adiponectin, soluble leptin-receptor, and HDL-cholesterol [57], FABP-4 may play a minor mediating role in the association between waist circumference and CRC risk in women.

The observed correlations of FABP-4 with a variety of biomarkers of inflammation, metabolism, blood lipids, adipokines, antioxidative capacity, and immune function (with strongest correlations observed for leptin and the antioxidant biomarker FRAP), some of which also have been previously associated with CRC risk, suggest that circulating FABP-4 is an integrative marker of various biological processes. Compared with the observed substantial correlations of FABP-4 with BMI and waist circumference, the weak positive correlation of FABP-4 with ABSI, a measure that was designed as a body shape index independent of BMI [37], suggests that FABP-4 is particularly influenced by general obesity.

Strengths of our investigation include the prospective study design, the ability to control for a variety of potential confounders in the biomarker analysis, and the use of MR enabling a further investigation of FABP-4 in relation to CRC risk circumventing certain types of bias common to studies of measured biomarkers. The three GWAS-identified SNPs included in the MR analysis were robustly associated with circulating FABP-4, but, together, explained only 1% of inter-individual variation. With an *F*-value of 65 (i.e., > 10, thereby not subject to weak instrument bias [48]), the first MR assumption (instrumental variable should be associated with the exposure) is satisfied although, with the given sample size, the statistical power was limited and we cannot exclude that small associations (RR < 1.17 per SD in FABP-4) have been missed. We assume that the second MR assumption (instrumental variables are independent of potential confounders) was fulfilled, although this could not be directly tested since we had no access to covariable data in the studies included in GECCO, CORECT, and CCFR. The third MR assumption (instrumental variable should be associated with the outcome only through the exposure of interest (FABP-4) — no horizontal pleiotropy) could also not be directly tested, but there was no indication of horizontal pleiotropy from MR Egger. Query of the phenoscanner database [58], however, revealed several adiposity and diabetes-related traits for the trans-SNP rs2012444, which seems plausible given the correlation of FABP-4 with body fatness measures and the previously observed association with type 2 diabetes [6], but suggests that this trans-SNP is associated besides FABP-4 with two established CRC risk factors, i.e., could be an invalid instrumental variable due to pleiotropic effects. Thus, the MR analysis using both cis and trans SNPs may have been subject to pleiotropy and should be interpreted cautiously. For the cis-SNPs (rs77878271 and rs2011042), no association with adiposity, diabetes, or other CRC risk factors has been reported, which strengthens the confidence in their use as instrumental variables. However, one trait related to mortality due to alcoholic hepatitis was listed for rs77878271. For the trans-SNP, rs79389622 as well as the cis-SNP, rs2011042, no associated traits besides FABP-4 were found. The stronger observed associations in MR using only the cis-SNP compared to all three (cis and trans) SNPs combined, may be explained by potential pleiotropy in the two trans-SNPs. Employing a cis-SNP in MR implies the highest biological plausibility [51]. Comparable to our results, a statistically significant association using only one cis-SNP as an instrumental variable, but statistically non-significant associations when using both the cis- and multiple trans-SNPs was observed in a MR study on IGF-1 and prostate cancer risk [59]. There was no sample overlap between the GWAS for FABP-4 (SCALLOP consortium) and the one for CRC (GECCO/CORECT/CCFR), which precludes inflated type one error rates [60].

A limitation of the biomarker analysis is that only a one-time FABP-4 measurement at baseline was available. Although the mid-term reliability (4 months) of FABP-4 has been shown to be relatively good [29], the one-time measurements may not necessarily reflect longer-term exposure. However, by the use of genetically determined FABP-4 in the MR, we were also able to investigate lifelong differences in FABP-4 in relation to CRC risk. Nevertheless, the results of the biomarker analysis could have been influenced by measurement error. Because the FABP-4 measurement was conducted on baseline samples before the onset of CRC, we expect such measurement error to be non-differential, which would not lead to biased estimates but could lead to attenuation of associations. Whereas the sample size of the biomarker analysis was sufficient to investigate CRC, it was limited for subgroup analysis by CRC anatomical subsite and sex. In terms of the MR analysis, another limitation was the relatively small sample size of the GWAS on FABP-4, which resulted in a limited number of SNPs independently associated with FABP-4, of which only three could be included in the present two-sample MR and these three SNPs explained only a small proportion of variability in FABP-4. Due to the limited genetic determination of FABP-4, the statistical power for the MR was slightly lower than in the biomarker analysis, and, again here, the sample size was limited for subgroup analyses by anatomical subsite and sex. In addition, the limited number of SNPs included in the present two-sample MR precluded detailed sensitivity analyses.

## Conclusions

This study combining data from several large epidemiological studies provides no strong support for a causal role of circulating FABP-4 in overall CRC risk, although the cis-MR provides some evidence for a positive association in women, which may deserve to be further investigated.

### Supplementary Information

**Additional file 1: Table S1.** Baseline characteristics by cases and controls. **Table S2.** Baseline characteristics by cases and controls, stratified by sex. **Table S3.** Baseline characteristics by sex in control participants. **Table S4.** Baseline characteristics by FABP4 quintiles in male controls. **Table S5.** Baseline characteristics by FABP4 quintiles in female controls. **Table S6.** Association of FABP-4 and risk of colorectal cancer with exclusion of people with diabetes and first 2 years of follow-up. **Table S7.** Association of FABP-4 and risk of colorectal cancer stratified by sex and subsite. **Table S8.** Colocalization analysis for FABP-4-colorectal cancer associations with prior probability *p* = 10^-5^. **Table S9.** Colocalization analysis for FABP-4-colorectal cancer associations with prior probability *p* = 10^-4^. **Figure S1.** Assumed directed acyclic graph (DAG) on potentially causal pathways in the association between FABP-4 and colorectal cancer risk and potentially confounding factors. **Figure S2.** Fixed-effects inverse variance–weighted Mendelian randomization analyses of FABP-4 and risk of colorectal cancer and its subsites. **Figure S3.** Cis Mendelian randomization analyses (Wald ratio) of FABP-4 and risk of colorectal cancer and its subsites. **Suppl. text 1.** GECCO Consortium funding.

## Data Availability

The EPIC data that support the findings of this study are available from the International Institute for Research on Cancer (IARC), but restrictions apply to the availability of these data, which were used under license for the current study, and so are not publicly available. Data are however available from the authors upon reasonable request and with permission of IARC. For information on how to submit an application for gaining access to EPIC data and/or bio-specimens, please follow the instructions at http://epic.iarc.fr/access/index.php. Summary data from the Genetics and Epidemiology of Colorectal Cancer Consortium (GECCO), Colorectal Cancer Transdisciplinary Study (CORECT), and Colon Cancer Family Registry (CCFR) are available upon request by contacting the respective studies.

## Abbreviations

ABSI: A-body shape index
BMI: Body mass index
CCFR: Colon Cancer Family Registry
CI: Confidence interval
CORECT: Colorectal Cancer Transdisciplinary Study
CRC: Colorectal cancer
EPIC: European Prospective Investigation into Cancer and Nutrition
FABP-4: Fatty acid binding protein-4
FRAP: Ferric reducing ability of plasma
GECCO: Genetics and Epidemiology of Colorectal Cancer Consortium
GWAS: Genome-wide association study
HbA1c: Glycated hemoglobin
HDL-C: High-density lipoprotein cholesterol
hsCRP: High-sensitivity C-reactive protein
IGF-1: Insulin-like growth factor 1
LDL-C: Low-density lipoprotein cholesterol
MR: Mendelian randomization
NAFLD: Non-alcoholic fatty liver disease
ROM: Reactive oxygen metabolites
RR: Relative risk
SD: Standard deviation
SNP: Single nucleotide polymorphism
sOB-R: Soluble leptin receptor
TNF-α: Tumor necrosis factor alpha
