# Supplementary material for: Prospective and Mendelian randomization analyses on the association of circulating fatty acid binding protein 4 (FABP-4) and risk of colorectal cancer
Source: BMC Med. 2023 Oct 13;21:391. doi: 10.1186/s12916-023-03104-1 (PMC10576353; doi:10.1186/s12916-023-03104-1)
Supplement: Supplementary file 1 — Additional file 1: Table S1. Baseline characteristics by cases and controls. Table S2. Baseline characteristics by cases and controls, stratified by sex. Table S3. Baseline characteristics by sex in control participants. Table S4. Baseline characteristics by FABP4 quintiles in male controls. Table S5. Baseline characteristics by FABP4 quintiles in female controls. Table S6. Association of FABP-4 and risk of colorectal cancer with exclusion of people with diabetes and first 2 years of follow-up. Table S7. Association of FABP-4 and risk of colorectal cancer stratified by sex and subsite. Table S8. Colocalization analysis for FABP-4-colorectal cancer associations with prior probability p = 10-5. Table S9. Colocalization analysis for FABP-4-colorectal cancer associations with prior probability p = 10-4. Figure S1. Assumed directed acyclic graph (DAG) on potentially causal pathways in the association between FABP-4 and colorectal cancer risk and potentially confounding factors. Figure S2. Fixed-effects inverse variance–weighted Mendelian randomization analyses of FABP-4 and risk of colorectal cancer and its subsites. Figure S3. Cis Mendelian randomization analyses (Wald ratio) of FABP-4 and risk of colorectal cancer and its subsites. Suppl. text 1. GECCO Consortium funding. [file 12916_2023_3104_MOESM1_ESM.docx]

**Prospective and Mendelian randomization analyses on the association of circulating fatty acid binding protein 4 (FABP-4) and risk of colorectal cancer**

Katharina Nimptsch, Krasimira Aleksandrova, Thu Thi Pham, Nikos Papadimitriou et al.

**Corresponding author:**

Dr. Katharina Nimptsch

Molecular Epidemiology Research Group

Max Delbrück Center for Molecular Medicine (MDC)

Robert-Rössle-Straße 10

13125 Berlin

Tel.: ++49 30 / 9406 – 4573

Fax: ++49 30 / 9406 – 4576

e-mail: katharina.nimptsch@mdc-berlin.de

**Additional file 1: Tables S1–S9, figures S1–S3, supplementary text 1.**

**Table S1 Baseline characteristics by cases and matched controls**

|  | Controls (n=1324) | Cases (n=1324) | p-value |
| --- | --- | --- | --- |
| Male, n (%) | 640 (48.3) | 640 (48.3) | * |
| Female, n (%) | 684 (51.7) | 684 (51.7) | * |
| Age, years | 58.1 (7.0) | 58.1 (7.0) | * |
| University degree, n (%) | 235 (17.7) | 227 (17.1) | 0.67 |
| Physically inactive, n (%) | 67 (5.1) | 110 (8.3) | 0.02 |
| Recreational and household physical activity, METs/week, median (IQR) | 78.5 (46.3,118.8) | 73.9 (44.6,116.0) | 0.13 |
| Smoker, n (%) | 328 (24.8) | 338 (25.5) | 0.64 |
| Body mass index, kg/m^2^, mean (SD) | 26.4 (3.8) | 26.8 (4.1) | 0.002 |
| Waist circumference, cm, mean (SD) | 88.7 (12.2) | 90.6 (12.8) | <0.0001 |
| A-body shape index, mean (SD) | 77.4 (5.7) | 78.1 (5.9) | 0.003 |
| Height, cm, mean (SD) | 167 (9.2) | 168 (9.1) | 0.02 |
| Diabetes at baseline, n (%) | 67 (5.1) | 110 (8.3) | 0.001 |
| Alcohol intake, g/day, median (IQR) | 8.0 (1.6, 22.4) | 8.7 (1.5, 24.2) | 0.10 |
| Dietary factors |  |  |  |
| Energy intake, kcal/day, median (IQR) | 2052 (1651, 2471) | 2076 (1688, 2504) | 0.72 |
| Fiber, g/day, median (IQR) | 23.0 (18.0, 27.9) | 22.0 (17.5, 27.2) | 0.01 |
| Fruits and vegetables, g/day, median (IQR) | 372.4 (246.5,547.0) | 365.8 (247.5,521.7) | 0.06 |
| Red meat, g/day, median (IQR) | 47.0 (25.1, 74.5) | 48.3 (25.4, 76.5) | 0.35 |
| Processed meat intake, g/day, median (IQR) | 25.1 (13.3, 44.3) | 25.5 (13.7, 44.5) | 0.20 |
| Fish, g/day, median (IQR) | 29.5 (14.7, 50.7) | 28.2 (15.1, 49.5) | 0.14 |
| FABP4, ng/mL, median (p25, p75) | 15.1 (11.0, 20.4) | 15.3 (11.1, 21.3) | 0.01 |

Mean (SD) unless indicated otherwise; SD, standard deviation; p25, 25^th^ percentile; p75, 75^th^ percentile

The p values for the difference between cases and controls based on McNemar’s test for variables expressed as %; Student’s paired t test for variables expressed as means; Wilcoxon’s signed rank test for variables expressed as medians.

* Matching variable

**Table S2 Baseline characteristics by cases and matched controls, stratified by sex**

|  | Men (n=1280) | |  | Women (n=1368) | |  |
| --- | --- | --- | --- | --- | --- | --- |
|  | Controls (n=640) | Cases (n=640) | p-value | Controls (n=684) | Cases (n=684) | p-value |
| Age, years | 58.1 (6.9) | 58.1 (6.9) | * | 58.1 (7.1) | 58.1 (7.1) | * |
| University degree, n (%) | 137 (21.4) | 134 (20.9) | 0.84 | 98 (14.3) | 93 (13.6) | 0.66 |
| Physically inactive, n (%) | 139 (21.7) | 145 (22.7) | 0.67 | 156 (22.8) | 197 (28.8) | 0.01 |
| Recreational and household physical activity, METs/week, median (IQR) | 59.8 (38.0, 89.7) | 55.1 (34.4, 85.4) | 0.25 | 99.1 (63.5,135.8) | 92.0 (59.6,134.6) | 0.34 |
| Smoker, n (%) | 187 (29.2) | 189 (29.5) | 0.90 | 141 (20.6) | 149 (21.8) | 0.57 |
| Body mass index, kg/m^2^, mean (SD) | 26.7 (3.4) | 27.4 (3.6) | 0.001 | 26.0 (4.1) | 26.3 (4.4) | 0.19 |
| Waist circumference, cm, mean (SD) | 95.7 (9.7) | 98.0 (9.9) | <0.0001 | 82.3 (10.6) | 83.8 (11.4) | 0.01 |
| A-body shape index, mean (SD) | 81.2 (3.9) | 81.8 (4.1) | 0.01 | 73.9 (4.8) | 74.8 (5.3) | 0.001 |
| Height, cm, mean (SD) | 174 (7.1) | 174 (6.9) | 0.16 | 161 (6.5) | 162 (6.6) | 0.06 |
| Diabetes at baseline, n (%) | 38 (5.9) | 67 (10.5) | 0.003 | 29 (4.2) | 43 (6.3) | 0.10 |
| Alcohol intake, g/day, median (p25, p75) | 13.7 (5.0, 34.5) | 16.2 (6.0, 41.0) | 0.02 | 4.4 (0.5, 12.6) | 4.0 (0.4, 12.2) | 0.60 |
| Dietary factors |  |  |  |  |  |  |
| Energy intake, kcal/day, median (p25, p75) | 2313 (1885, 2745) | 2301 (1920, 2742) | 0.85 | 1855 (1517, 2196) | 1873 (1564, 2213) | 0.44 |
| Fiber, g/day, median (p25, p75) | 24.1 (18.6, 29.5) | 22.9 (18.0, 28.4) | 0.01 | 22.2 (17.7, 26.3) | 21.4 (17.2, 26.2) | 0.31 |
| Fruits and vegetables, g/day, median (p25, p75) | 336.0 (190.0,516.7) | 328.3 (216.3,473.2) | 0.10 | 410.3 (282.9,578.0) | 405.0 (280.4,557.2) | 0.31 |
| Red meat, g/day, median (p25, p75 | 52.3 (27.0, 84.5) | 57.0 (28.6, 91.0) | 0.12 | 41.5 (22.6, 65.8) | 42.3 (22.6, 64.5) | 0.80 |
| Processed meat intake, g/day, median (p25, p75) | 33.0 (18.3, 53.0) | 34.5 (18.2, 58.0) | 0.21 | 19.4 (10.2, 33.6) | 20.4 (11.3, 34.0) | 0.67 |
| Fish, g/day, median (IQR) | 32.2 (16.6, 54.2) | 31.2 (16.4, 54.5) | 0.20 | 26.2 (11.6, 47.5) | 25.3 (13.6, 44.4) | 0.48 |
| FABP4, ng/mL, median (p25, p75) | 12.1 (9.0, 16.0) | 12.4 (9.0, 16.2) | 0.41 | 18.3 (14.0, 24.5) | 19.3 (14.5, 25.5) | 0.01 |

Mean (SD) unless indicated otherwise; SD, standard deviation; p25, 25^th^ percentile; p75, 75^th^ percentile

The p values for the difference between cases and controls based on McNemar’s test for variables expressed as %; Student’s paired t test for variables expressed as means; Wilcoxon’s signed rank test for variables expressed as medians.

* Matching variable

**Table S3. Baseline characteristics by sex in control participants (n=1324)**

|  | Men (n=640) | Women (n=684) | p-value |
| --- | --- | --- | --- |
| Age, years | 58.1 (6.9) | 58.1 (7.1) | 0.92 |
| University degree, n (%) | 137 (21.4) | 98 (14.3) | 0.001 |
| Physically inactive, n (%) | 139 (21.7) | 156 (22.8) | 0.63 |
| Recreational and household physical activity, METs/week, median (p25, p75) | 59.8 (38.0, 89.7) | 99.1 (63.5,135.8) | <0.0001 |
| Smoker, n (%) | 187 (29.2) | 141 (20.6) | 0.0003 |
| Body mass index, kg/m^2^, mean (SD) | 26.7 (3.4) | 26.0 (4.1) | 0.001 |
| Waist circumference, cm, mean (SD) | 95.7 (9.7) | 82.3 (10.6) | <0.0001 |
| A-body shape index, mean (SD) | 81.2 (78.7, 83.6) | 73.8 (70.8, 76.9) | <0.0001 |
| Height, cm, mean (SD) | 174 (7.1) | 161 (6.5) | <0.0001 |
| Diabetes at baseline, n (%) | 24 (3.8) | 23 (3.4) | 0.33 |
| Alcohol intake, g/day, median (p25, p75) | 13.7 (5.0, 34.5) | 4.4 (0.5, 12.6) | <0.0001 |
| Dietary factors |  |  |  |
| Energy intake, kcal/day, median (p25, p75) | 2313 (1885, 2745) | 1855 (1517, 2196) | <0.0001 |
| Fiber, g/day, median (p25, p75) | 24.1 (18.6, 29.5) | 22.2 (17.7, 26.3) | <0.0001 |
| Fruits and vegetables, g/day, median (p25, p75) | 336.0 (190.0,516.7) | 410.3 (282.9,578.0) | <0.0001 |
| Red meat, g/day, median (p25, p75) | 33.0 (18.3, 53.0) | 19.4 (10.2, 33.6) | <0.0001 |
| Processed meat intake, g/day, median (p25, p75) | 52.3 (27.0, 84.5) | 41.5 (22.6, 65.8) | <0.0001 |
| Fish, g/day, median (p25, p75) | 32.2 (16.6, 54.2) | 26.2 (11.6, 47.5) | <0.0001 |
| FABP4, ng/mL, median (p25, p75) | 12.1 (9.0, 16.0) | 18.3 (14.0, 24.5) | <0.0001 |

SD, standard deviation; p25, 25^th^ percentile; p75, 75^th^ percentile

P-values from analysis of variance for variables expressed as means, Chi^2^-test for variables expressed as % and Kruskal-Wallis non-parametric test for variables expressed as median

**Table S4. Baseline characteristics by (sex-specific) quintiles of FABP4 concentrations in male control participants (n=640)**

|  | Quintile 1 | Quintile 2 | Quintile 3 | Quintile 4 | Quintile 5 | p-trend |
| --- | --- | --- | --- | --- | --- | --- |
| Quintile ranges in men | <8.3 ng/mL | 8.3-<10.8 ng/mL | 10.8-<13.6 ng/mL | 13.6-<17.2 ng/mL | ≥17.2 ng/mL |  |
| N | 128 | 128 | 128 | 128 | 128 |  |
| Age, years | 56.6 (6.9) | 58.6 (6.6) | 57.1 (7.5) | 58.9 (6.4) | 59.2 (6.7) | 0.01 |
| University degree, n (%) | 24 (18.8) | 26 (20.3) | 34 (26.6) | 30 (23.4) | 23 (18.0) | 0.89 |
| Physically inactive, n (%) | 19 (14.8) | 23 (18.0) | 36 (28.1) | 29 (22.7) | 32 (25.0) | 0.03 |
| Recreational and household physical activity, METs/week, median (p25, p75) | 83.5 (48.5,123.2) | 78.9 (49.9,118.7) | 76.1 (42.6,115.6) | 75.7 (48.6,115.5) | 75.2 (42.0,123.0) | 0.58 |
| Smoker, n (%) | 36 (28.1) | 33 (25.8) | 32 (25.0) | 46 (35.9) | 40 (31.3) | 0.20 |
| Body mass index, kg/m^2^, mean (SD) | 25.1 (3.0) | 26.0 (2.8) | 26.5 (2.7) | 27.4 (3.2) | 28.7 (3.9) | <0.0001 |
| Waist circumference, cm, mean (SD) | 90.5 (9.1) | 94.4 (8.9) | 95.1 (7.5) | 97.4 (9.6) | 102 (9.9) | <0.0001 |
| A-body shape index, mean (SD) | 80.1 (3.9) | 81.6 (4.5) | 81.1 (3.8) | 81.2 (3.4) | 82.4 (3.5) | 0.0003 |
| Height, cm, mean (SD) | 174 (7.1) | 174 (6.8) | 173 (7.2) | 174 (7.7) | 174 (6.7) | 0.72 |
| Diabetes at baseline, n (%) | 7 (5.5) | 9 (7.0) | 5 (3.9) | 7 (5.5) | 10 (7.8) | 0.27 |
| Alcohol intake, g/day, median (p25, p75) | 16.5 (6.4, 36.3) | 14.7 (4.8, 35.2) | 12.0 (3.4, 31.4) | 16.1 (6.7, 36.9) | 10.8 (2.9, 30.6) | 0.05 |
| Dietary factors |  |  |  |  |  |  |
| Energy intake, kcal/day, median (p25, p75) | 2357 (2003, 2812) | 2331 (1957, 2808) | 2296 (1770, 2657) | 2340 (1962, 2791) | 2195 (1762, 2627) | 0.08 |
| Fiber, g/day, median (p25, p75) | 25.6 (19.9, 32.0) | 24.9 (19.0, 31.7) | 22.7 (17.9, 28.6) | 24.2 (18.6, 28.2) | 23.0 (17.9, 27.4) | 0.01 |
| Fruits and vegetables, g/day, median (p25, p75) | 365.5 (192.3,537.3) | 400.7 (261.7,625.3) | 305.2 (192.2,475.2) | 310.0 (190.0,471.9) | 290.0 (165.8,426.4) | 0.00 |
| Red meat, g/day, median (p25, p75) | 34.1 (16.8, 54.5) | 33.9 (14.9, 52.7) | 30.6 (16.9, 52.8) | 32.9 (20.6, 51.6) | 35.7 (20.4, 54.6) | 0.57 |
| Processed meat intake, g/day, median (p25, p75) | 48.6 (24.0, 78.7) | 47.1 (25.7, 84.4) | 52.5 (26.3, 82.5) | 59.2 (40.3, 85.4) | 55.5 (25.8, 95.8) | 0.09 |
| Fish, g/day, median (p25, p75) | 33.9 (15.9, 62.7) | 32.2 (16.4, 50.2) | 29.4 (16.5, 47.4) | 33.9 (17.5, 57.6) | 33.0 (17.2, 53.7) | 0.65 |
| FABP4, ng/mL, median (p25, p75) | 6.8 (5.5, 7.5) | 9.6 (9.0, 10.2) | 12.1 (11.4, 12.8) | 15.2 (14.4, 16.0) | 20.0 (18.4, 23.7) | <0.0001 |

FABP-4, fatty acid binding protein 4; SD, standard deviation; p25, 25^th^ percentile; p75, 75^th^ percentile

P for trend across quintiles from generalized linear model for variables expressed as means, from Jonkheere-Terpstra test for variables expressed as percentage, from Kruskal-Wallis test for variables expressed as median

**Table S5. Baseline characteristics by (sex-specific) quintiles of FABP4 concentrations in female control participants (n=684)**

|  | Quintile 1 | Quintile 2 | Quintile 3 | Quintile 4 | Quintile 5 | p-trend |
| --- | --- | --- | --- | --- | --- | --- |
| Quintile ranges in women | <13.0 ng/mL | 13.0-<16.8 ng/mL | 16.8-<20.5 ng/mL | 20.5-<26.3 ng/mL | ≥26.33 ng/mL |  |
| N | 135 | 140 | 138 | 137 | 134 |  |
| Age, years | 54.8 (7.0) | 56.7 (7.5) | 59.3 (7.2) | 59.3 (6.1) | 60.4 (6.4) | <0.0001 |
| University degree, n (%) | 24 (17.8) | 30 (21.4) | 16 (11.6) | 11 (8.0) | 17 (12.7) | 0.01 |
| Physically inactive, n (%) | 21 (15.6) | 27 (19.3) | 37 (26.8) | 32 (23.4) | 39 (29.1) | 0.01 |
| Recreational and household physical activity, METs/week, median (IQR) | 83.5 (48.5,123.2) | 78.9 (49.9,118.7) | 76.1 (42.6,115.6) | 75.7 (48.6,115.5) | 75.2 (42.0,123.0) | 0.58 |
| Smoker, n (%) | 39 (28.9) | 28 (20.0) | 25 (18.1) | 23 (16.8) | 26 (19.4) | 0.04 |
| Body mass index, kg/m^2^, mean (SD) | 23.1 (2.8) | 24.4 (3.2) | 26.5 (3.9) | 27.4 (3.3) | 28.7 (4.6) | <0.0001 |
| Waist circumference, cm, mean (SD) | 75.3 (7.6) | 78.2 (8.0) | 83.8 (10.5) | 85.2 (8.2) | 89.7 (11.7) | <0.0001 |
| A-body shape index, mean (SD) | 73.0 (4.7) | 73.2 (4.6) | 74.4 (5.1) | 74.0 (4.4) | 75.3 (4.8) | <0.0001 |
| Height, cm, mean (SD) | 162 (7.1) | 162 (7.0) | 160 (6.0) | 161 (6.6) | 161 (5.9) | 0.03 |
| Diabetes at baseline, n (%) | 3 (2.2) | 2 (1.4) | 7 (5.1) | 8 (5.8) | 9 (6.7) | 0.02 |
| Alcohol intake, g/day, median (p25, p75) | 6.5 (0.5, 13.2) | 4.1 (0.6, 11.7) | 3.9 (0.5, 12.0) | 4.8 (0.4, 13.5) | 3.8 (0.6, 12.5) | 0.89 |
| Dietary factors |  |  |  |  |  |  |
| Energy intake, kcal/day, median (p25, p75) | 1873 (1525, 2250) | 1872 (1488, 2212) | 1827 (1576, 2143) | 1890 (1521, 2234) | 1810 (1451, 2201) | 0.76 |
| Fiber, g/day, median (p25, p75) | 22.8 (18.2, 27.8) | 21.5 (16.9, 25.7) | 22.9 (18.2, 26.3) | 21.8 (17.6, 25.9) | 22.4 (16.6, 25.9) | 0.42 |
| Fruits and vegetables, g/day, median (p25, p75) | 441.8 (280.8,610.1) | 394.2 (291.6,525.9) | 418.6 (286.7,628.9) | 427.5 (290.9,587.8) | 388.3 (260.1,561.8) | 0.54 |
| Red meat, g/day, median (p25, p75) | 18.3 (10.8, 35.3) | 20.7 (10.9, 30.0) | 19.0 (11.0, 33.6) | 20.4 (9.4, 36.8) | 17.2 (9.6, 31.1) | 0.83 |
| Processed meat intake, g/day, median (p25, p75) | 38.8 (23.6, 64.8) | 39.8 (18.4, 67.9) | 41.5 (21.8, 62.4) | 42.8 (24.2, 65.6) | 45.5 (24.2, 67.7) | 0.82 |
| Fish, g/day, median (p25, p75) | 22.5 (12.3, 44.0) | 20.4 (10.2, 44.9) | 28.0 (14.1, 49.3) | 22.9 (8.9, 45.4) | 32.2 (14.0, 50.1) | 0.13 |
| FABP4, ng/mL, median (p25, p75) | 11.2 (8.8, 12.1) | 14.7 (14.0, 15.7) | 18.3 (17.7, 19.3) | 23.5 (22.2, 24.6) | 30.9 (28.8, 36.5) | <0.0001 |

FABP-4, fatty acid binding protein 4; SD, standard deviation; p25, 25^th^ percentile; p75, 75^th^ percentile

P for trend across quintiles from generalized linear model for variables expressed as means, from Jonkheere-Terpstra test for variables expressed as percentage, from Kruskal-Wallis test for variables expressed as median **Table S6. Association between baseline FABP-4 concentrations and risk of colorectal cancer (conditional logistic regression models), with exclusion of people with diabetes and first 2 years of follow-up**

|  | Without exclusion (as in table 3) | | | People with diabetes excluded | | | First 2 years of follow-up excluded | | |
| --- | --- | --- | --- | --- | --- | --- | --- | --- | --- |
|  | Ca/Co | OR^§^ | (95% CI) | Ca/Co | OR^§^ | (95% CI) | Ca/Co | OR^§^ | (95% CI) |
|  | *Overall (1324 case-control pairs)* | | | *Overall (1153 case-control pairs)* | | | *Overall (1089 case-control pairs)* | | |
| Quintile 1 | 237/263 | 1 | Reference | 219/231 | 1 | Reference | 190/215 | 1 | Reference |
| Quintile 2 | 256/268 | 1.08 | (0.84,1.40) | 226/241 | 0.98 | (0.75,1.29) | 219/213 | 1.20 | (0.90,1.60) |
| Quintile 3 | 268/266 | 1.14 | (0.88,1.48) | 248/232 | 1.11 | (0.84,1.46) | 212/219 | 1.11 | (0.83,1.49) |
| Quintile 4 | 267/265 | 1.12 | (0.86,1.45) | 227/229 | 1.01 | (0.77,1.33) | 224/226 | 1.13 | (0.85,1.51) |
| Quintile 5 | 296/262 | 1.26 | (0.96,1.66) | 233/220 | 1.07 | (0.80,1.43) | 244/216 | 1.28 | (0.95,1.73) |
| p-trend |  |  | 0.08 |  |  | 0.59 |  |  | 0.12 |
| per SD of FABP-4 (8.9 ng/ml)* | | 1.09 | (0.99,1.19) |  | 1.03 | (0.94,1.13) |  | 1.09 | (0.99,1.20) |
|  | *Men (640 case-control pairs)* | | | *Men (541 case-control pairs)* | | | *Men (516 case-control pairs)* | | |
| Quintile 1 | 118/128 | 1 | Reference | 103/106 | 1 |  | 94/98 | 1 |  |
| Quintile 2 | 119/128 | 1 | (0.68,1.46) | 99/110 | 0.87 | (0.57,1.32) | 98/99 | 1.02 | (0.67,1.56) |
| Quintile 3 | 139/128 | 1.21 | (0.83,1.75) | 126/110 | 1.15 | (0.77,1.72) | 108/105 | 1.03 | (0.68,1.58) |
| Quintile 4 | 127/128 | 1.04 | (0.71,1.52) | 107/111 | 0.93 | (0.62,1.39) | 107/106 | 1.00 | (0.65,1.52) |
| Quintile 5 | 137/128 | 1.11 | (0.74,1.64) | 106/104 | 0.96 | (0.62,1.47) | 109/108 | 0.96 | (0.62,1.49) |
| p-trend |  |  | 0.65 |  |  | 0.87 |  |  | 0.79 |
| per SD of FABP-4 (8.9 ng/ml)* | | 1.07 | (0.92,1.23) |  | 1.03 | (0.89,1.20) |  | 1.01 | (0.86,1.19) |
|  | *Women (684 case-control pairs)* | | | *Women (612 case-control pairs)* | | | *Women (573 case-control pairs)* | | |
| Quintile 1 | 119/135 | 1 | Reference | 116/125 | 1 |  | 96/117 | 1 |  |
| Quintile 2 | 137/140 | 1.14 | (0.80,1.64) | 127/131 | 1.06 | (0.73,1.53) | 121/114 | 1.4 | (0.94,2.09) |
| Quintile 3 | 129/138 | 1.12 | (0.77,1.64) | 122/122 | 1.1 | (0.74,1.64) | 104/114 | 1.24 | (0.81,1.89) |
| Quintile 4 | 140/137 | 1.22 | (0.84,1.79) | 120/118 | 1.1 | (0.74,1.63) | 117/120 | 1.33 | (0.88,2.01) |
| Quintile 5 | 159/134 | 1.46 | (0.99,2.17) | 127/116 | 1.2 | (0.79,1.83) | 135/108 | **1.76** | **(1.14,2.71)** |
| p-trend |  |  | 0.05 |  |  | 0.39 |  |  | **0.03** |
| per SD of FABP-4 (8.9 ng/ml)* | | **1.12** | **(1.00,1.26)** |  | 1.05 | (0.92,1.19) |  | **1.17** | **(1.03,1.34)** |
| p-heterogeneity by sex | | | 0.57 |  |  | 0.39 |  |  | 0.21 |

FABP-4, fatty acid binding protein 4; Ca/Co, numbers of cases/controls; RR, relative risk based on estimated incidence rate ratio; CI, confidence interval; SD, standard deviation (SD calculated in controls)

^§^ Conditioned on matching factors (sex, age at blood collection, study center, time of blood collection and fasting status; in women were additionally matched on menopausal status, phase of the menstrual cycle among premenopausal women, and use of hormone replacement therapy at the time of blood collection among postmenopausal women) and adjusted for education (none, primary school, technical/professional or secondary school, longer education including university degree, not specified), physical activity index (inactive, moderately inactive, moderately active, active, missing), smoking status and intensity (never, current (1-15, 16-25, 26+ cig/day, pipe/cigars/occasionally, intensity missing), former (quit ≤10, 11-20, 20+ years ago, unknown), alcohol intake (nondrinker, former drinker, current drinker, current g/day at baseline).

* SD calculated in controls

**Table S7. Association between baseline FABP-4 concentrations and risk of colorectal cancer (conditional logistic regression models), stratified by sex and subsite**

|  | N Case-control pairs | RR^†^ | (95% CI) | RR^‡^ | (95% CI) | RR^§^ | (95% CI) |
| --- | --- | --- | --- | --- | --- | --- | --- |
| *Colon cancer, overall* | 829 | 1.08 | (0.97,1.20) | 1.06 | (0.95,1.18) | 0.96 | (0.85,1.09) |
| *Colon, men* | 372 | 1.03 | (0.87,1.22) | 1.05 | (0.87,1.27) | 0.89 | (0.69,1.15) |
| *Colon, women* | 457 | 1.10 | (0.96,1.26) | 1.11 | (0.96,1.27) | 1.06 | (0.89,1.26) |
| p-heterogeneity by sex for colon cancer |  |  | 0.60 |  | 0.67 |  | 0.27 |
|  |  |  |  |  |  |  |  |
| *Proximal colon, overall* | *355* | 1.04 | (0.90,1.19) | 1.04 | (0.90,1.21) | 0.95 | (0.81,1.12) |
| *Proximal colon, men* | 153 | 1.03 | (0.84,1.26) | 1.12 | (0.86,1.46) | 1.03 | (0.78,1.38) |
| *Proximal colon, women* | 202 | 1.05 | (0.86,1.27) | 1.12 | (0.90,1.39) | 1.04 | (0.81,1.34) |
| p-heterogeneity by sex for proximal colon cancer |  |  | 0.91 |  | 0.60 |  | 0.97 |
| *Distal colon, overall* | 409 | 1.16 | (0.97,1.37) | 1.13 | (0.94,1.36) | 0.94 | (0.75,1.16) |
| p-heterogeneity (competing risk) proximal versus distal colon |  |  | 0.14 |  | 0.59 |  | 0.80 |
| *Distal colon, men* | 184 | 1.08 | (0.78,1.49) | 0.97 | (0.66,1.41) | 0.64 | (0.40,1.02) |
| p-heterogeneity (competing risk) proximal versus distal colon in men |  |  | 0.21 |  | 0.89 |  | 0.87 |
| *Distal colon, women* | 225 | 1.19 | (0.97,1.46) | 1.19 | (0.96,1.49) | 1.11 | (0.85,1.43) |
| p-heterogeneity by sex for distal colon cancer |  |  | 0.62 |  | 0.34 |  | 0.05 |
| p-heterogeneity (competing risk) proximal versus distal colon in women |  |  | 0.33 |  | 0.26 |  | 0.37 |
|  |  |  |  |  |  |  |  |
| *Rectum, overall* | 478 | 1.16 | (1.00,1.36) | 1.16 | (0.99,1.37) | 1.12 | (0.93,1.36) |
| p-heterogeneity (competing risk) colon versus rectum |  |  | <0.0001 |  | 0.0002 |  | 0.35 |
| *Rectum, men* | 257 | 1.13 | (0.89,1.44) | 1.09 | (0.82,1.45) | 1.00 | (0.73,1.37) |
| p-heterogeneity (competing risk) colon versus rectum in men |  |  | <0.0001 |  | 0.09 |  | 0.73 |
| *Rectum, women* | 221 | 1.19 | (0.97,1.46) | 1.19 | (0.94,1.50) | 1.20 | (0.91,1.58) |
| p-heterogeneity by sex for rectal cancer |  |  | 0.74 |  | 0.66 |  | 0.39 |
| p-heterogeneity (competing risk) colon versus rectum in women |  |  | <0.0001 |  | <0.0001 |  | 0.97 |

FABP-4, fatty acid binding protein 4; CI, confidence interval; SD, standard deviation;

RR, relative risk based on estimated incidence rate ratio, per standard deviation (SD) in FABP-4 (8.9 ng/ml), SD calculated in controls

^†^ conditioned on matching factors (sex, age at blood collection, study center, time of blood collection and fasting status; in women were additionally matched on menopausal status, phase of the menstrual cycle among premenopausal women, and use of hormone replacement therapy at the time of blood collection among postmenopausal women)

^‡^ additionally adjusted for education (none, primary school, technical/professional or secondary school, longer education including university degree, not specified), physical activity index (inactive, moderately inactive, moderately active, active, missing), smoking status and intensity (never, current (1-15, 16-25, 26+ cig/day, pipe/cigars/occasionally, intensity missing), former (quit <=10, 11-20, 20+ years ago, unknown), alcohol intake (nondrinker, former drinker, current drinker, current g/day at baseline)

^§^ additionally adjusted for BMI, height and BMI- and height- adjusted waist circumference residuals

**Table S8. Colocalization analysis for FABP-4-colorectal cancer associations with prior probability p=10^-5^**

|  | PP1  *causal variant for FABP-4 only* | PP2  *causal variant for CRC only* | PP3  *two distinct causal variants* | PP4  *one shared causal variant* |
| --- | --- | --- | --- | --- |
| CRC, overall | 0.98 | 1.21E-11 | 5.46E-04 | 0.02 |
| CRC, men | 0.99 | 1.23E-11 | 5.65E-04 | 0.01 |
| CRC, women | 0.88 | 4.56E-11 | 2.02E-03 | 0.12 |

PP, posterior probability

**Table S9. Colocalization analysis for FABP-4-colorectal cancer associations with prior probability p=10^-4^**

|  | PP1  *causal variant for FABP-4 only* | PP2  *causal variant for CRC only* | PP3  *two distinct causal variants* | PP4  *one shared causal variant* |
| --- | --- | --- | --- | --- |
| CRC, overall | 0.82 | 1.01E-11 | 4.55E-04 | 0.19 |
| CRC, men | 0.90 | 1.11E-11 | 5.13E-04 | 0.10 |
| CRC, women | 0.42 | 2.17E-11 | 9.63E-04 | 0.58 |

PP, posterior probability


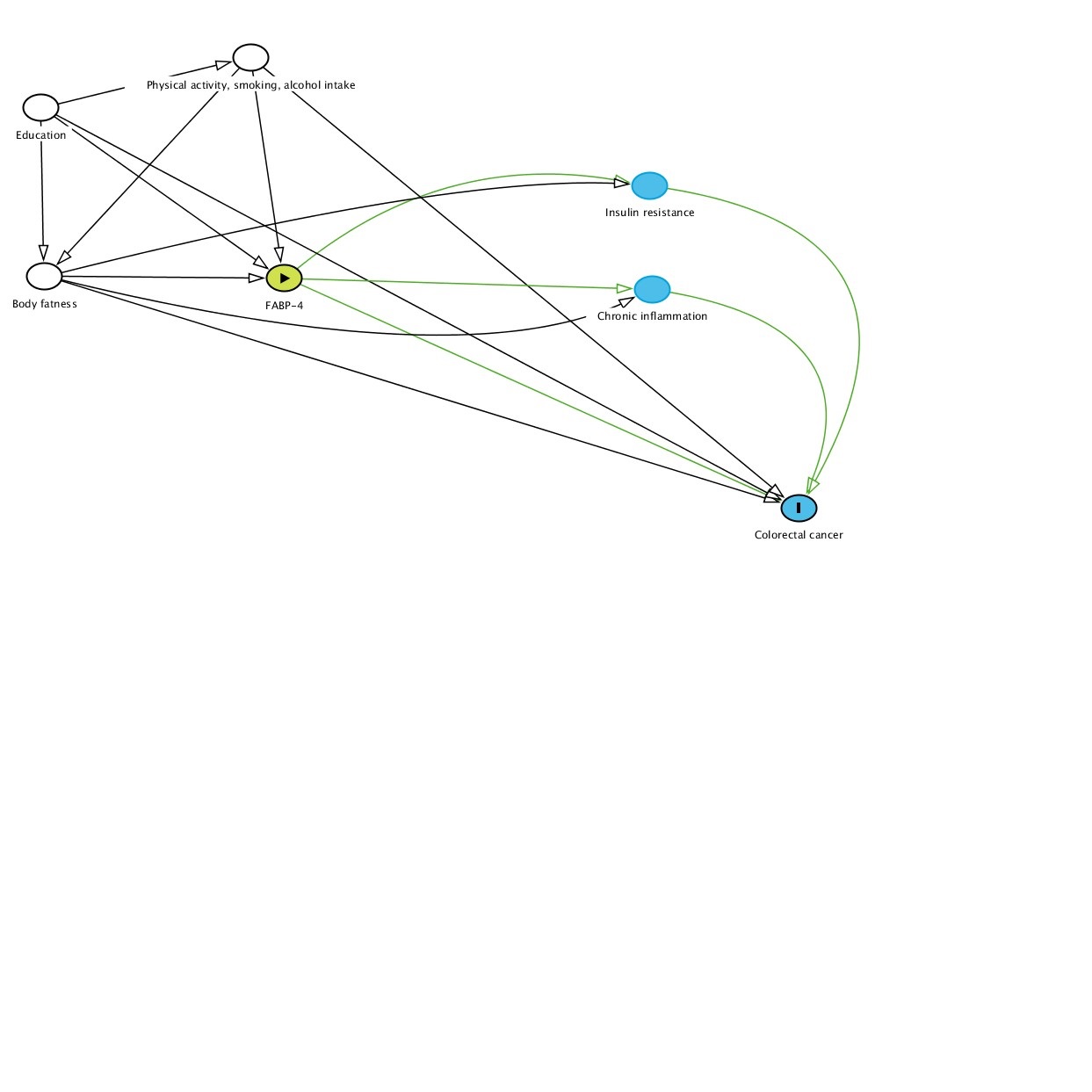


**Figure S1.** **Assumed directed acyclic graph (DAG) on potentially causal pathways in the association between FABP-4 and colorectal cancer risk and potentially confounding factors (matching factors are not included in the diagram)**


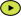
 exposure

outcome


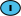


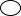
 adjusted variable


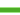
 (potentially) causal path

Reference and software: Textor J, van der Zander B, Gilthorpe MS, Liskiewicz M, Ellison GT. Robust causal inference using directed acyclic graphs: the R package 'dagitty'. Int J Epidemiol. 2016;45(6):1887-94.

**Figure S2.** **Fixed-effects inverse variance–weighted Mendelian randomization analyses of FABP-4 and risk of colorectal cancer and its subsites based on a 2-sample MR with SNP-FABP-4 associations for three SNPs (one cis, two trans) from SCALLOP consortium and SNP-CRC associations from GECCO, CORECT and CCFR**

OR, odds ratio, CI, confidence interval; FABP-4, fatty acid binding protein 4; MR, Mendelian Randomization

**Figure S3.** **Mendelian randomization analyses (Wald ratio) of FABP-4 and risk of colorectal cancer and its subsites based on a 2-sample MR with SNP-FABP-4 associations for one cis-SNP from SCALLOP consortium and SNP-CRC associations from GECCO, CORECT and CCFR**

OR, odds ratio, CI, confidence interval; FABP-4, fatty acid binding protein 4; MR, Mendelian Randomization

**Supplementary text 1.** **GECCO Consortium funding**

Genetics and Epidemiology of Colorectal Cancer Consortium (GECCO): National Cancer Institute, National Institutes of Health, U.S. Department of Health and Human Services (U01 CA137088, R01 CA059045, R01 201407). Genotyping/Sequencing services were provided by the Center for Inherited Disease Research (CIDR) contract number HHSN268201700006I and HHSN268201200008I . This research was funded in part through the NIH/NCI Cancer Center Support Grant P30 CA015704. Scientific Computing Infrastructure at Fred Hutch funded by ORIP grant S10OD028685.

ASTERISK: a Hospital Clinical Research Program (PHRC-BRD09/C) from the University Hospital Center of Nantes (CHU de Nantes) and supported by the Regional Council of Pays de la Loire, the Groupement des Entreprises Françaises dans la Lutte contre le Cancer (GEFLUC), the Association Anne de Bretagne Génétique and the Ligue Régionale Contre le Cancer (LRCC).

The ATBC Study is supported by the Intramural Research Program of the U.S. National Cancer Institute, National Institutes of Health, Department of Health and Human Services.

CLUE II funding was from the National Cancer Institute (U01 CA086308, Early Detection Research Network; P30 CA006973), National Institute on Aging (U01 AG018033), and the American Institute for Cancer Research. The content of this publication does not necessarily reflect the views or policies of the Department of Health and Human Services, nor does mention of trade names, commercial products, or organizations imply endorsement by the US government.

Maryland Cancer Registry (MCR)

Cancer data was provided by the Maryland Cancer Registry, Center for Cancer Prevention and Control, Maryland Department of Health, with funding from the State of Maryland and the Maryland Cigarette Restitution Fund. The collection and availability of cancer registry data is also supported by the Cooperative Agreement NU58DP006333, funded by the Centers for Disease Control and Prevention. Its contents are solely the responsibility of the authors and do not necessarily represent the official views of the Centers for Disease Control and Prevention or the Department of Health and Human Services.

ColoCare: This work was supported by the National Institutes of Health (grant numbers R01 CA189184 (Li/Ulrich), U01 CA206110 (Ulrich/Li/Siegel/Figueiredo/Colditz, 2P30CA015704- 40 (Gilliland), R01 CA207371 (Ulrich/Li)), the Matthias Lackas-Foundation, the German Consortium for Translational Cancer Research, and the EU TRANSCAN initiative.

The Colon Cancer Family Registry (CCFR, www.coloncfr.org) is supported in part by funding from the National Cancer Institute (NCI), National Institutes of Health (NIH) (award U01 CA167551). Support for case ascertainment was provided in part from the Surveillance, Epidemiology, and End Results (SEER) Program and the following U.S. state cancer registries: AZ, CO, MN, NC, NH; and by the Victoria Cancer Registry (Australia) and Ontario Cancer Registry (Canada). The CCFR Set-1 (Illumina 1M/1M-Duo) and Set-2 (Illumina Omni1-Quad) scans were supported by NIH awards U01 CA122839 and R01 CA143237 (to GC). The CCFR Set-3 (Affymetrix Axiom CORECT Set array) was supported by NIH award U19 CA148107 and R01 CA81488 (to SBG). The CCFR Set-4 (Illumina OncoArray 600K SNP array) was supported by NIH award U19 CA148107 (to SBG) and by the Center for Inherited Disease Research (CIDR), which is funded by the NIH to the Johns Hopkins University, contract number HHSN268201200008I. Additional funding for the OFCCR/ARCTIC was through award GL201-043 from the Ontario Research Fund (to BWZ), award 112746 from the Canadian Institutes of Health Research (to TJH), through a Cancer Risk Evaluation (CaRE) Program grant from the Canadian Cancer Society (to SG), and through generous support from the Ontario Ministry of Research and Innovation. The SFCCR Illumina HumanCytoSNP array was supported in part through NCI/NIH awards U01/U24 CA074794 and R01 CA076366 (to PAN). The content of this manuscript does not necessarily reflect the views or policies of the NCI, NIH or any of the collaborating centers in the Colon Cancer Family Registry (CCFR), nor does mention of trade names, commercial products, or organizations imply endorsement by the US Government, any cancer registry, or the CCFR.

COLON: The COLON study is sponsored by Wereld Kanker Onderzoek Fonds, including funds from grant 2014/1179 as part of the World Cancer Research Fund International Regular Grant Programme, by Alpe d’Huzes and the Dutch Cancer Society (UM 2012–5653, UW 2013-5927, UW2015-7946), and by TRANSCAN (JTC2012-MetaboCCC, JTC2013-FOCUS). The Nqplus study is sponsored by a ZonMW investment grant (98-10030); by PREVIEW, the project PREVention of diabetes through lifestyle intervention and population studies in Europe and around the World (PREVIEW) project which received funding from the European Union Seventh Framework Programme (FP7/2007–2013) under grant no. 312057; by funds from TI Food and Nutrition (cardiovascular health theme), a public–private partnership on precompetitive research in food and nutrition; and by FOODBALL, the Food Biomarker Alliance, a project from JPI Healthy Diet for a Healthy Life.

COLO2&3: National Institutes of Health (R01 CA060987).

Colorectal Cancer Transdisciplinary (CORECT) Study: The CORECT Study was supported by the National Cancer Institute, National Institutes of Health (NCI/NIH), U.S. Department of Health and Human Services (grant numbers U19 CA148107, R01 CA081488, P30 CA014089, R01 CA197350; P01 CA196569; R01 CA201407; R01 CA242218), National Institutes of Environmental Health Sciences, National Institutes of Health (grant number T32 ES013678) and a generous gift from Daniel and Maryann Fong.

CORSA: The CORSA study was funded by Austrian Research Funding Agency (FFG) BRIDGE (grant 829675, to Andrea Gsur), the “Herzfelder’sche Familienstiftung” (grant to Andrea Gsur) and was supported by COST Action BM1206.

CPS-II: The American Cancer Society funds the creation, maintenance, and updating of the Cancer Prevention Study-II (CPS-II) cohort. The study protocol was approved by the institutional review boards of Emory University, and those of participating registries as required.

CRCGEN: Colorectal Cancer Genetics & Genomics, Spanish study was supported by Instituto de Salud Carlos III, co-funded by FEDER funds –a way to build Europe– (grants PI14-613 and PI09-1286), Agency for Management of University and Research Grants (AGAUR) of the Catalan Government (grant 2017SGR723), Junta de Castilla y León (grant LE22A10-2), the Spanish Association Against Cancer (AECC) Scientific Foundation grant GCTRA18022MORE and the Consortium for Biomedical Research in Epidemiology and Public Health (CIBERESP), action Genrisk. Sample collection of this work was supported by the Xarxa de Bancs de Tumors de Catalunya sponsored by Pla Director d’Oncología de Catalunya (XBTC), Plataforma Biobancos PT13/0010/0013 and ICOBIOBANC, sponsored by the Catalan Institute of Oncology. We thank CERCA Programme, Generalitat de Catalunya for institutional support.

Czech Republic CCS: This work was supported by the Grant Agency of the Czech Republic (21-04607X, 20-03997S), by the Grant Agency of the Ministry of Health of the Czech Republic (grants AZV NU21-07-00247 and AZV NU21-03-00506), and Charles University Research Fund (Cooperation 43-Surgical disciplines)

DACHS: This work was supported by the German Research Council (BR 1704/6-1, BR 1704/6-3, BR 1704/6-4, CH 117/1-1, HO 5117/2-1, HE 5998/2-1, KL 2354/3-1, RO 2270/8-1 and BR 1704/17-1), the Interdisciplinary Research Program of the National Center for Tumor Diseases (NCT), Germany, and the German Federal Ministry of Education and Research (01KH0404, 01ER0814, 01ER0815, 01ER1505A and 01ER1505B).

DALS: National Institutes of Health (R01 CA048998 to M. L. Slattery).

EDRN: This work is funded and supported by the NCI, EDRN Grant (U01-CA152753).

EPIC: The coordination of EPIC is financially supported by International Agency for Research on Cancer (IARC) and also by the Department of Epidemiology and Biostatistics, School of Public Health, Imperial College London which has additional infrastructure support provided by the NIHR Imperial Biomedical Research Centre (BRC). The national cohorts are supported by: Danish Cancer Society (Denmark); Ligue Contre le Cancer, Institut Gustave Roussy, Mutuelle Générale de l’Education Nationale, Institut National de la Santé et de la Recherche Médicale (INSERM) (France); German Cancer Aid, German Cancer Research Center (DKFZ), German Institute of Human Nutrition Potsdam- Rehbruecke (DIfE), Federal Ministry of Education and Research (BMBF) (Germany); Associazione Italiana per la Ricerca sul Cancro-AIRC-Italy, Compagnia di SanPaolo and National Research Council (Italy); Dutch Ministry of Public Health, Welfare and Sports (VWS), Netherlands Cancer Registry (NKR), LK Research Funds, Dutch Prevention Funds, Dutch ZON (Zorg Onderzoek Nederland), World Cancer Research Fund (WCRF), Statistics Netherlands (The Netherlands); Health Research Fund (FIS) - Instituto de Salud Carlos III (ISCIII), Regional Governments of Andalucía, Asturias, Basque Country, Murcia and Navarra, and the Catalan Institute of Oncology - ICO (Spain); Swedish Cancer Society, Swedish Research Council and and Region Skåne and Region Västerbotten (Sweden); Cancer Research UK (14136 to EPIC-Norfolk; C8221/A29017 to EPIC-Oxford), Medical Research Council (1000143 to EPIC-Norfolk; MR/M012190/1 to EPIC-Oxford). (United Kingdom).

EPICOLON: This work was supported by grants from Fondo de Investigación Sanitaria/FEDER (PI08/0024, PI08/1276, PS09/02368, P111/00219, PI11/00681, PI14/00173, PI14/00230, PI17/00509, 17/00878, PI20/00113, PI20/00226, Acción Transversal de Cáncer), Xunta de Galicia (PGIDIT07PXIB9101209PR), Ministerio de Economia y Competitividad (SAF07-64873, SAF 2010-19273, SAF2014-54453R), Fundación Científica de la Asociación Española contra el Cáncer (GCB13131592CAST), Beca Grupo de Trabajo “Oncología” AEG (Asociación Española de Gastroenterología), Fundación Privada Olga Torres, FP7 CHIBCHA Consortium, Agència de Gestió d’Ajuts Universitaris i de Recerca (AGAUR, Generalitat de Catalunya, 2014SGR135, 2014SGR255, 2017SGR21, 2017SGR653), Catalan Tumour Bank Network (Pla Director d’Oncologia, Generalitat de Catalunya), PERIS (SLT002/16/00398, Generalitat de Catalunya), CERCA Programme (Generalitat de Catalunya) and COST Action BM1206 and CA17118. CIBERehd is funded by the Instituto de Salud Carlos III.

ESTHER/VERDI. This work was supported by grants from the Baden-Württemberg Ministry of Science, Research and Arts and the German Cancer Aid.

Harvard cohorts: HPFS is supported by the National Institutes of Health (P01 CA055075, UM1 CA167552, U01 CA167552, R01 CA137178, R01 CA151993, and R35 CA197735), NHS by the National Institutes of Health (P01 CA087969, UM1 CA186107, R01 CA137178, R01 CA151993, and R35 CA197735), and PHS by the National Institutes of Health (R01 CA042182).

Hawaii Adenoma Study: NCI grants R01 CA072520.

HCES-CRC: the Hwasun Cancer Epidemiology Study–Colon and Rectum Cancer (HCES-CRC; grants from Chonnam National University Hwasun Hospital, HCRI15011-1).

Kentucky: This work was supported by the following grant support: Clinical Investigator Award from Damon Runyon Cancer Research Foundation (CI-8); NCI R01CA136726.

LCCS: The Leeds Colorectal Cancer Study was funded by the Food Standards Agency and Cancer Research UK Programme Award (C588/A19167).

MCCS cohort recruitment was funded by VicHealth and Cancer Council Victoria. The MCCS was further supported by Australian NHMRC grants 509348, 209057, 251553 and 504711 and by infrastructure provided by Cancer Council Victoria. Cases and their vital status were ascertained through the Victorian Cancer Registry (VCR) and the Australian Institute of Health and Welfare (AIHW), including the National Death Index and the Australian Cancer Database. BMLynch was supported by MCRF18005 from the Victorian Cancer Agency.

MEC: National Institutes of Health (R37 CA054281, P01 CA033619, and R01 CA063464).

MECC: This work was supported by the National Institutes of Health, U.S. Department of Health and Human Services (R01 CA081488, R01 CA197350, U19 CA148107, R01 CA242218, and a generous gift from Daniel and Maryann Fong.

MSKCC: The work at Sloan Kettering in New York was supported by the Robert and Kate Niehaus Center for Inherited Cancer Genomics and the Romeo Milio Foundation. Moffitt: This work was supported by funding from the National Institutes of Health (grant numbers R01 CA189184, P30 CA076292), Florida Department of Health Bankhead-Coley Grant 09BN-13, and the University of South Florida Oehler Foundation. Moffitt contributions were supported in part by the Total Cancer Care Initiative, Collaborative Data Services Core, and Tissue Core at the H. Lee Moffitt Cancer Center & Research Institute, a National Cancer Institute-designated Comprehensive Cancer Center (grant number P30 CA076292).

NCCCS I & II: We acknowledge funding support for this project from the National Institutes of Health, R01 CA066635 and P30 DK034987.

NFCCR: This work was supported by an Interdisciplinary Health Research Team award from the Canadian Institutes of Health Research (CRT 43821); the National Institutes of Health, U.S. Department of Health and Human Serivces (U01 CA074783); and National Cancer Institute of Canada grants (18223 and 18226). The authors wish to acknowledge the contribution of Alexandre Belisle and the genotyping team of the McGill University and Génome Québec Innovation Centre, Montréal, Canada, for genotyping the Sequenom panel in the NFCCR samples. Funding was provided to Michael O. Woods by the Canadian Cancer Society Research Institute.

NSHDS: The research was supported by Biobank Sweden through funding from the Swedish Research Council (VR 2017-00650, VR 2017-01737), the Swedish Cancer Society (CAN 2017/581), Region Västerbotten (VLL-841671, VLL-833291), Knut and Alice Wallenberg Foundation (VLL-765961), and the Lion’s Cancer Research Foundation (several grants) and Insamlingsstiftelsen, both at Umeå University.

OSUMC: OCCPI funding was provided by Pelotonia and HNPCC funding was provided by the NCI (CA016058 and CA067941).

PLCO: Intramural Research Program of the Division of Cancer Epidemiology and Genetics and supported by contracts from the Division of Cancer Prevention, National Cancer Institute, NIH, DHHS. Funding was provided by National Institutes of Health (NIH), Genes, Environment and Health Initiative (GEI) Z01 CP 010200, NIH U01 HG004446, and NIH GEI U01 HG 004438.

SEARCH: The University of Cambridge has received salary support in respect of PDPP from the NHS in the East of England through the Clinical Academic Reserve. Cancer Research UK (C490/A16561); the UK National Institute for Health Research Biomedical Research Centres at the University of Cambridge.

SELECT: Research reported in this publication was supported in part by the National Cancer Institute of the National Institutes of Health under Award Numbers U10 CA037429 (CD Blanke), and UM1 CA182883 (CM Tangen/IM Thompson). The content is solely the responsibility of the authors and does not necessarily represent the official views of the National Institutes of Health.

SM and REACHS: This work was supported by the National Cancer Institute (grant P01 CA074184 to J.D.P. and P.A.N., grants R01 CA097325, R03 CA153323, and K05 CA152715 to P.A.N., and the National Center for Advancing Translational Sciences at the National Institutes of Health (grant KL2 TR000421 to A.N.B.-H.)

The Swedish Low-risk Colorectal Cancer Study: The study was supported by grants from the Swedish research council; K2015-55X-22674-01-4, K2008-55X-20157-03-3, K2006-72X-20157-01-2 and the Stockholm County Council (ALF project).

Swedish Mammography Cohort and Cohort of Swedish Men: This work is supported by the Swedish Research Council /Infrastructure grant, the Swedish Cancer Foundation, and the Karolinska Institute´s Distinguished Professor Award to Alicja Wolk.

UK Biobank: This research has been conducted using the UK Biobank Resource under Application Number 8614

VITAL: National Institutes of Health (K05 CA154337).

WHI: The WHI program is funded by the National Heart, Lung, and Blood Institute, National Institutes of Health, U.S. Department of Health and Human Services through contracts HHSN268201100046C, HHSN268201100001C, HHSN268201100002C, HHSN268201100003C, HHSN268201100004C, and HHSN271201100004C.
